# Learning from a lot: Empirical Bayes for high‐dimensional model‐based prediction

**DOI:** 10.1111/sjos.12335

**Published:** 2018-06-01

**Authors:** Mark A. van de Wiel, Dennis E. Te Beest, Magnus M. Münch

**Affiliations:** ^1^ Department of Epidemiology and Biostatistics, Amsterdam Public Health Research Institute VU University Medical Center Amsterdam The Netherlands; ^2^ Department of Mathematics VU University Amsterdam The Netherlands; ^3^ Mathematical Institute, Faculty of Science Leiden University Leiden The Netherlands

**Keywords:** co‐data, empirical Bayes, marginal likelihood, prediction, variable selection

## Abstract

Empirical Bayes is a versatile approach to “learn from a lot” in two ways: first, from a large number of variables and, second, from a potentially large amount of prior information, for example, stored in public repositories. We review applications of a variety of empirical Bayes methods to several well‐known model‐based prediction methods, including penalized regression, linear discriminant analysis, and Bayesian models with sparse or dense priors. We discuss “formal” empirical Bayes methods that maximize the marginal likelihood but also more informal approaches based on other data summaries. We contrast empirical Bayes to cross‐validation and full Bayes and discuss hybrid approaches. To study the relation between the quality of an empirical Bayes estimator and p, the number of variables, we consider a simple empirical Bayes estimator in a linear model setting. We argue that empirical Bayes is particularly useful when the prior contains multiple parameters, which model a priori information on variables termed “co‐data”. In particular, we present two novel examples that allow for co‐data: first, a Bayesian spike‐and‐slab setting that facilitates inclusion of multiple co‐data sources and types and, second, a hybrid empirical Bayes–full Bayes ridge regression approach for estimation of the posterior predictive interval.

## 1 INTRODUCTION

High‐dimensional data with tens or hundreds of thousands of variables are frequently part of biomedical (or other) studies nowadays. In addition, a lot of prior information is available in the public domain, for example, in genomics data repositories or in data bases containing structural information on the variables such as genomic pathways. When one aims to develop a predictor for a new study, one is challenged to learn from this wealth of data. For many high‐dimensional prediction methods, such learning consists of two phases: 1) learning the tuning parameter(s) like, for example, penalty parameters in a frequentist framework or prior parameters in a Bayesian framework; and 2) learning the predictor as a function of the variables given the tuning parameter(s). Empirical Bayes (EB) is a widely acknowledged approach to complete the first phase.

Broadly speaking, EB is a collection of methods that estimate the tuning parameter(s), often formulated in terms of prior parameters, from the data, thereby borrowing information across variables of the same type. We focus mostly on high‐dimensional prediction settings, so p>n, with p as the number of predictors and n as the number of independent samples. For other settings, several excellent contributions exist. Carlin and Louis (2000) is an extensive introduction to EB. It discusses parametric and nonparametric EB, provides many examples for standard models, presents suggestions on computations (in particular, for maximization of the marginal likelihood), and compares performances of EB methods with fully Bayesian and frequentist ones in low‐dimensional settings. Efron (2010) has quickly become a standard work for applications of Empirical Bayes to multiple testing, particularly for estimation of the false discovery rate and variants thereof. Van Houwelingen (2014) is a recent critical review with many data examples on the application of EB to low‐dimensional estimation problems, particularly in meta‐analysis, and to high‐dimensional multiple testing problems. Some of the pros and cons of EB mentioned in these references are reiterated here, but cast in the perspective of high‐dimensional prediction. Note that the properties and usefulness of EB estimators may be different in high‐ and low‐dimensional prediction settings. First, high‐dimensional data allows for more complex, possibly sparse priors with several hyperparameters. Moreover, the computational advantage (with respect to full Bayes) is larger in high‐dimensional settings. In addition, the large p may lead to better estimation of the prior (see Sections 2.1 and 5.2) and allows for modeling the prior in terms of prior information on the variables (see Section 6). Finally, regularization changes the bias–variance tradeoff and hence the properties of the EB estimator (see Section 5.2).

While the emphasis in this paper is on high‐dimensional prediction, we sometimes refer to the “medium‐dimensional” setting. The latter is informally defined as a p<n setting but with p large enough (with respect to n) to render estimation ill‐behaved and hence desire regularization. We believe that this medium‐dimensional prediction setting is becoming increasingly relevant. For example, targeted high‐throughput molecular devices have become cheaper, implying that their use for clinical prediction has become realistic. These devices typically measure tens or hundreds of molecular markers, possibly selected from “whole‐genome” screening studies. Examples of such devices are multiplex polymerase chain reaction (PCR) and targeted sequencing platforms.

We review several versions of EB plus their applications to a variety of prediction methods. We follow Morris (1983): “Empirical Bayes modeling permits statisticians to incorporate additional information in problems” and argue that such prior information on the variables, referred to as “co‐data” (see also Neuenschwander, Roychoudhury, & Schmidli, 2016), is particularly useful in high‐dimensional settings because it may improve prediction and variable selection. Such co‐data may be continuous, for example, p‐values from a related but independent study or nominal, for example, known sets of variables that share a function. The use of such co‐data to accommodate different priors for variables is known as “local adaptation” in full Bayes settings (O'Hara & Sillanpää, 2009); we discuss the EB counterpart.

When the unknown hyperparameter(s) concern tuning parameters in a frequentist setting, cross‐validation is a popular alternative for EB. Therefore, we contrast the two approaches and shortly discuss hybrid solutions. We cannot cover the entire scala of high‐dimensional prediction methods and hence focus on model‐based prediction. We do discuss a fairly broad spectrum, including penalized regression (e.g., lasso, ridge, and elastic net), linear discriminant analysis, and Bayesian approaches using sparse or dense priors. Ridge regression is used to illustrate matters on several occasions, particularly to analytically study the expected mean squared error of an empirical Bayes estimator of the prior variance as a function of p. Although the theory on EB in large p settings is an active field of research, results are still mostly limited to very simple models as discussed in Section 4.

Finally, we present two novel examples of the usage of EB for high‐ and medium‐dimensional prediction. The two examples both allow to account for co‐data when estimating the prior(s). The first example illustrates how EB may be used to inform prior inclusion probabilities in a Bayesian spike‐and‐slab model that is fit using Markov chain Monte Carlo (MCMC) sampling. Second, a simulation example demonstrates the benefit of a hybrid Bayes–EB approach for the estimation of the posterior predictive interval using group‐regularized logistic ridge regression.

## 2 EMPIRICAL BAYES METHODOLOGIES

We review several EB methodologies in the context of model‐based high‐dimensional prediction. Their applicability depends on the prediction method, which we will specify in the sections below. We distinguish:

1. MMLU EB: maximize the marginal likelihood product derived from univariate models.
2. MMLJ EB: maximize marginal likelihood derived from a joint model.
  - Direct EB: maximize an analytical expression for the marginal likelihood.
  - Laplace EB: maximize marginal likelihood using Laplace approximation.
  - MCMC EB: maximize marginal likelihood using MCMC sampling.
  - VB EB: maximize marginal likelihood using Variational Bayes (VB).
3. MoM EB: method of moments; equate theoretical moments to empirical ones.

Several fundamental similarities and differences across 1) to 3) exist. First, the use of 1) is restricted to prediction methods that combine univariate models into one prediction such as diagonal linear discriminant analysis. We show that marginal likelihood‐based empirical Bayes, which shrinks the effect sizes, is then very similar to “standard” empirical Bayes in estimation problems. Methodology 2) applies formal EB to the full multivariate setting, hence to a single joint p‐dimensional model like, penalized regression. As such, it is the most generic methodology. The methodology is then subclassified by methods that are used to facilitate the maximization, the suitability of which depends on the prediction model used. Finally, 3) refers to an intuitive classical use of EB: equating moments. Naturally, this is restricted to predictors for which the moments are known. Below, we provide details on 1) to 3).

Throughout this article, we denote response by **Y**=(Y_1_,…,Y_n_) and the high‐dimensional parameter by **θ**=(θ_1_,…,θ_p_). Variables are denoted by $X={\left(X_{1}^{T},\text{…},X_{n}^{T}\right)}^{T},X_{i}=(X_{i1},\text{…},X_{ip})$.

### 2.1 Maximum marginal likelihood from univariate models: MMLU EB

One of the simplest classifiers that may be used in a high‐dimensional setting is diagonal linear discriminant analysis (DLDA). It assumes a diagonal covariance matrix Σ for the variables. While this is unlikely to be true, the results of DLDA may be better than that of ordinary LDA, which requires a (regularized) estimate of Σ (Bickel & Levina, 2004). One of the early classifiers introduced for high‐dimensional prediction, the shrunken centroid algorithm (Tibshirani, Hastie, Narasimhan, & Chu, 2002), may be regarded as a DLDA. Moreover, DLDA is discussed here because EB for DLDA turns out to be very similar to EB for estimation problems, allowing a gentle introduction to EB. Here, we follow the notation of Efron (2009); DLDA combines univariate effect–size estimates ${\hat{\theta }}_{j}$ in one classification rule by the sign of S_i_, with

(1) $$S_{i}=\sum_{j=1}^{p}{\hat{\theta }}_{j}W_{ij},$$

where W_ij_ is the standardized value of variable X_ij_. For this type of classifier, EB‐type shrinkage is based on univariate summaries as in many multiple testing and estimation settings. Following Efron (2009), we compute the Z‐score Z_j_, which is the standardized difference in means between the two groups (defined by **Y**) for variable j. Then, Z_j_ is expressed as a convolution

(2) $$Z_{j}=\theta_{j}+\epsilon_{j},$$

where ϵ_j_∼N(0,1) and θ_j_∼π, with assumptions $\epsilon_{j}⫫\theta_{j}$ and $Z_{j}⫫Z_{k}$, for k≠j. Efron (2009) then continues by developing a nonparametric estimate of π using deconvolution, and this could in fact be regarded as a form of nonparametric EB. The posterior mean, ${\hat{\theta }}_{j}=E(\theta_{j}\;|\;Z_{j})$, then provides a shrunken estimate of θ_j_. Dicker and Zhao (2016) use a very similar marginal nonparametric deconvolution approach. Their work is based on the Bayes classifier and provides theoretical guarantees on the performance if the deconvolution is accurate and the joint densities of the two groups of **X**_i_ variables are far apart in terms of Hellinger distance.

Here, we discuss the parametric counterpart, meaning π=π_**α**_ is of a specified parametric form with unknown hyperparameters **α**. This could be useful when one would desire a sparse DLDA, requiring a sparse prior, for example, a spike‐and‐slab prior. In the parametric setting, estimating **α** then boils down to maximizing the (marginal) likelihood, which factorizes rendering

(3) $$\hat{\alpha }={\text{argmax}}_{\alpha }\left(\prod_{j=1}^{p}\int_{\theta_{j}}L(Z_{j};\theta_{j})\pi_{\alpha }(\theta_{j})d\theta_{j}\right)={\text{argmax}}_{\alpha }\log\left(\sum_{j=1}^{p}\int_{\theta_{j}}L(Z_{j};\theta_{j})\pi_{\alpha }(\theta_{j})d\theta_{j}\right),$$

where $L(Z_{j};\theta_{j})$ is the (Gaussian) likelihood implied by (2). The maximization of (3) is relatively straightforward because the integral is one‐dimensional. In case the prior is conjugate, it may be solved analytically; otherwise, efficient EM‐type algorithms are available such as the one in van de Wiel et al. (2012), which was proven to converge. Once $\hat{\alpha }$ and hence $\pi_{\hat{\alpha }}$ are known, the computation of the shrunken estimate ${\hat{\theta }}_{j}=E(\theta_{j}\;|\;Z_{j};\hat{\alpha })$ is straightforward; substitution into (1) then renders the (possibly sparse) DLDA.

Note that convolution (2) and EB estimate (3) are exactly the same as in the well‐known normal means estimation problem. This problem is well studied and theoretically understood (Johnstone & Silverman, 2004). In the Supporting Information, we revisit the famous batting averages example (Efron & Morris, 1975), which is a normal means problem that is often used as a scholarly example of EB estimation. It concerns the data of 18 baseball players. Van Houwelingen (2014) rightfully criticizes EB in this setting because it seems to overshrink the estimate for the best player(s) when using a Gaussian prior. We show that, when one would have had additional data of 10,000 players, the overshrinkage is much less severe because the EB estimate of the Gaussian prior variance improves a lot. This connects to what we will observe in Section 5 for the linear ridge regression model. For the enlarged batting data, the large p also accommodates the use of a more complex prior, like, a three‐component Gaussian mixture, which slightly further reduces the shrinkage for the extremes.

The likelihood product in (3) contrasts the marginal likelihood corresponding to joint prediction models. The latter contains a high‐dimensional integral over **θ**, and is thus much more complex. Given that the vast majority of statistical prediction methods are based on joint models, we now turn our attention to those.

### 2.2 Maximum marginal likelihood from a joint model: MMLJ EB

Suppose that we wish to use a prediction method on the basis of a joint prediction model that implies likelihood L(Y;θ). For convenience, variables **X**, which are usually part of L(Y;θ) via regression, are not denoted in it. Then, an empirical Bayes estimate is obtained by maximizing the marginal likelihood:

(4) $$\hat{\alpha }={\text{argmax}}_{\alpha }\text{ML}(\alpha ),\;\text{with}\;\text{ML}(\alpha )=\int_{\theta }L(Y;\theta )\pi_{\alpha }(\theta )d\theta ,$$

with prior π_**α**_(**θ**). Often, the prior is assumed to have a product form: $\pi_{\alpha }(\theta )=\prod_{j=1}^{p}\pi_{\alpha }(\theta_{j})$. While marginal likelihood is a Bayesian concept, (4) may also be used in penalized regression settings because of the correspondence between **α** and the penalty parameter(s), say **λ**, in the penalized likelihood. A well‐known example is the elastic net, with ridge and lasso as special cases (Zou & Hastie, 2005). Below, we discuss several methods to solve (4).

#### 2.2.1 Direct EB

If the prior is conjugate to the likelihood in (4), computations highly simplify because this enables the direct maximization of the marginal likelihood. For example, for the linear regression model with a shared Gaussian prior θ_j_∼N(0,τ ^2^) and Gaussian error variance σ ^2^, that is, ridge regression, we have

$$\text{ML}(\alpha )=\text{ML}\left(\left(\tau^{2},\sigma^{2}\right)\right)=N\left(Y;\mu =0,\Sigma =XX^{T}\tau^{2}+I_{n\times n}\sigma^{2}\right),$$

which allows for straightforward likelihood maximization. This directly renders an estimator of the ridge penalty: $\hat{\lambda }={\hat{\sigma }}^{2}/{\hat{\tau }}^{2}$, which is computationally more efficient than cross‐validation. Ridge regression is basically a random effects model and hence fits in the setting of mixed models. Such models may include fixed effects as well, useful for accommodating covariates like age or known biomarkers in a clinical prediction model. Jiang, Li, Debashis, Yang, and Zhao (2016a) discuss the well‐known restricted maximum likelihood (REML) estimator of (τ ^2^,σ ^2^). They prove the consistency of the REML estimator in the high‐dimensional setting, even when the prior is misspecified, in the sense that only a fraction of regression parameters are nonzero in reality.

Karabatsos (2017) extends the direct MML estimation to a Bayesian generalized ridge model (using a flat gamma prior on σ ^−2^), which allows differential penalization of the principal components of **X**. This setting includes the power ridge as a special case, implying a multivariate Gaussian prior with covariance matrix τ ^2^(**X** ^T^ **X**)^δ^, where δ is an additional hyperparameter and τ ^2^=σ ^2^/λ. Karabatsos (2017) presents a two‐stage algorithm to maximize the ML with respect to δ and λ.

In many prediction problems, conjugacy is not achieved either because of the nature of the response **Y** (e.g., binary or survival) or the nature of the (preferred) prior (e.g., a sparse prior). Then, alternative solutions are needed. Below, we present two of these, both of which are more generic than MMLU EB and direct EB.

#### 2.2.2 Laplace EB

In nonconjugate settings, the high‐dimensional integral in (4) poses a major difficulty, preventing a direct analytical solution. Hence, approximations have been developed for ML(**α**) for various choices of the likelihood and the prior, particularly for penalized regression with regression parameters **θ**. The integrand of (4) can often be reformulated in an exponential form, motivating the use of Laplace approximations:

(5) $$\int_{\theta }e^{-nh_{\alpha }(\theta )}d\theta \approx e^{-nh_{\alpha }\left(\hat{\theta }\right)}{\left(2\pi \right)}^{p/2}\det\;{\left(H_{\alpha }^{-1}\right)}^{1/2}n^{-p/2},$$

where **H**_**α**_ is the Hessian of h_**α**_(**θ**), evaluated at $\hat{\theta }$. Usually, $\hat{\theta }={\text{argmax}}_{\theta }\;h_{\alpha }(\theta )$ is used. This maximum depends on the unknown **α**. For many priors efficient maximizers of the integrand of (4) and hence h_**α**_(**θ**) exist. This suggests numerical optimization or EM‐type algorithms alternating between maximization with respect to **θ** given **α** and Laplace approximation plus maximization in terms of **α**. An example is given in Heisterkamp, van Houwelingen, and Downs (1999) for a Poisson model with Gaussian priors.

Concerns have been raised about the accuracy of (5) in high‐dimensional settings. For example, Shun and McCullagh (1995) suggest that when p>O(n ^1/3^), the standard Laplace approximation may be unreliable. Sparse priors, which effectuate variable selection, may render approximation (5) to be accurate but only when the prior is “sparse enough”. Intuitively, a sparse prior may render the effective dimension of the integral of (5) much smaller than p, because $\hat{\theta }$ contains many zeros. Barber, Drton, and Tan (2016) consider the Laplace approximation to the marginal likelihood of Bayesian generalized linear models with sparse selection priors of the form

Pν(J)∝p|J|−ν1{|J|≤q},J⊂{1,…,p},

where J is the set of selected variables (i.e., nonzero θ_j_'s), q is a maximum of selected variables, and ν is a tuning parameter. Here, ν determines whether the prior distribution of the models (ν=0), or the prior distribution of the model cardinalities (ν=1) is uniform. They show that with q relatively small (sparse setting) and sample size sufficiently large, the Laplace approximation to the marginal likelihood can be accurate for a potentially large number of models, implying that it may be employed for the estimation of hyperparameters in strongly sparse settings.

Apart from the accuracy of the Laplace approximation, another issue is that h_**α**_(**θ**) in (5) may not have a second derivative, rendering the Hessian undefined. An example is regression with a Laplace prior, known as the Bayesian lasso. The L_1_‐norm on **θ** is not differentiable at zero with respect to the θ_j_ and can therefore not be approximated by the Laplace method without modifications.

#### 2.2.3 Markov chain Monte Carlo EB

If Laplace approximation to the integral in the right‐hand side of (4) is not possible or feasible, we may circumvent explicit calculation by an MCMC sampler. Desired quantities are easily calculated from these samples. Casella (2001) proves that one may employ an EM algorithm to estimate the hyperparameters from Gibbs samples. The algorithm was extended to general MCMC sampling by Levine and Casella (2001) who also provide an approximation of the Monte Carlo error. The algorithm is an MCEM‐type algorithm (Wei & Tanner, 1990) based on posterior samples of **θ** instead of point estimates. Here, we shortly describe the method. First, write the marginal likelihood as

(6) $$\text{ML}(\alpha )=\frac{L(Y,\theta ;\alpha )}{p(\theta \;|\;Y;\alpha )},$$

where L(Y,θ;α) and p(**θ** | **Y**;**α**) denote the conditional likelihood of **α** (i.e., the joint distribution of **Y** and **θ** for fixed **α**) and posterior distribution of the model parameters, respectively. We take the expectation of both sides with respect to p(**θ** | **Y**;**α** ′) and switch to the log scale to arrive at

(7) $$E_{\alpha^{\prime }}[\log\text{ML}(\alpha )]=E_{\alpha^{\prime }}[\ell (Y,\theta ;\alpha )]-E_{\alpha^{\prime }}[\log p(\theta \;|\;Y;\alpha )]$$

for some (current value) **α** ′. Expand the last term of (7):

$$E_{\alpha^{\prime }}[\log p(\theta \;|\;Y;\alpha )]=\int \log p(\theta \;|\;Y;\alpha )p(\theta \;|\;Y;\alpha^{\prime })d\theta$$

and note that by Gibbs' inequality this integral is maximized at **α**=**α** ′. Consequently, for every **α**≠**α** ′, $-E_{\alpha^{\prime }}[\log p(\theta \;|\;Y;\alpha^{\prime })]<-E_{\alpha^{\prime }}[\log p(\theta \;|\;Y;\alpha )]$, such that the sequence, which iteratively maximizes the first term in the right‐hand side of (7)

(8) $$\alpha^{\left(k+1\right)}={\text{argmax}}_{\alpha }E_{\alpha^{\left(k\right)}}[\ell (Y,\theta ;\alpha )],$$

is nondecreasing and converges. The expectation in (8) will generally not be available in closed form. However, one may approximate it by its Monte Carlo estimate:

(9) $${\text{argmax}}_{\alpha }E_{\alpha^{\left(k\right)}}[\ell (Y,\theta ;\alpha )]\approx {\text{argmax}}_{\alpha }\frac{1}{M}\sum_{m=1}^{M}\ell (Y,\theta^{m,(k)};\alpha ),$$

where **θ** ^m,(k)^ denotes the mth MCMC sample from the posterior distribution with hyperparameters **α** ^(k)^ and ℓ(**Y**,**θ** ^m,(k)^;**α**) is the conditional log‐likelihood of **α** evaluated at the mth MCMC sample. Often, ℓ(**Y**,**θ** ^m,(k)(m)^;**α**) has a fairly simple tractable form as exemplified in Section 6.1. Applications of this method are the estimation of the penalty parameter(s) for the Bayesian lasso in Park and Casella (2008) and for the Bayesian elastic net in Li and Lin (2010). In a penalized logistic regression setting, the efficient Gibbs sampler described in Polson, Scott, and Windle (2013) may be used.

The method above is very generic: It may be applied for hyperparameter estimation using, in principle, any Bayesian sampling technique. It is computationally costly though: the EM iterations require multiple MCMC updates, although the number of runs can be reduced by periodically alternating with updates from an importance sampling approximation (Casella, 2001). To limit Monte Carlo error of the marginal log‐likelihood estimate in (9), the MCMC sample size should be sufficiently large. Booth and Hobert (1999) propose to start with small sample sizes and increase the sample size as long as the expected likelihood is “swamped” by Monte Carlo error. The small initial sample size is justified with the EM algorithm's tendency to take large steps towards the optimum in the first few iterations. Any Monte Carlo error in the log‐likelihood estimate is relatively small compared to the large increase in log‐likelihood during these iterations. Close to convergence, the EM algorithm tends to increment the log‐likelihood in smaller steps. Then, the Monte Carlo error is relatively larger, requiring a larger sample size to counteract this. For some models, the MCMC sample size may be reduced by introducing stochastic approximation in the E‐step (Kuhn & Lavielle, 2004). In Section 6.1, we illustrate how to apply MCMC EB to high‐dimensional spike‐and‐slab models and show that it straightforwardly allows to moderate the inclusion prior by use of co‐data.

#### 2.2.4 Variational Bayes–EB

For some models, variational Bayes (VB) approximations (for a review, see Blei, Kucukelbir, & McAuliffe, 2017) can be developed as a very efficient alternative to MCMC, which is also useful in the EM algorithm above. Moreover, VB lends itself well for EB estimation because the nature of the approximation often allows expressing the expectation in (8) analytically in terms of **α**. Let us assume a simple hierarchical model: **Y**←**θ**←**Z**←**α**. For example, the Bayesian lasso (Park & Casella, 2008) has **θ** as p‐dimensional regression parameter, **Z** is the p‐dimensional latent mixture parameter in a scale mixture of normals, and **α**=λ_1_ is the lasso penalty parameter. Nuisance parameters like error variance σ ^2^ may be added w.l.o.g.

Let p(**θ**,**Z** | **Y**;**α**) denote the full posterior. In the context of our model, VB approximation amounts to determining functions q_1_ and q_2_ such that q_1_(**θ**)q_2_(**Z**;**α**) minimizes the Kullback–Leibner distance KL(q_1_ q_2_||p). Finding solutions $q_{1}^{*}$ and $q_{2}^{*}$ requires specific derivations for the model at hand. Several are available in the literature such as for spike‐and‐slab regression (Carbonetto & Stephens, 2012), the Bayesian ridge model (Leday et al., 2017), and the Bayesian lasso (Joo, 2017). For example, in the latter model $q_{1}^{*}$ is a multivariate Gaussian, whereas $q_{2}^{*}$ conveniently factorizes with respect to Z_1_,…,Z_p_ as a product of inverse Gaussians.

The VB analogue of the MCMC EB algorithm above is then straightforward: in the EM algorithm above, replace the Monte Carlo approximation of the posterior, required for the expected joint likelihood (9), by the VB approximation $q_{1}^{*}(\theta )q_{2}^{*}(Z;\alpha )$. In the hierarchical model setting, maximization w.r.t. **α** then amounts to computing the posterior mean of the log‐prior of **Z**

(10) $$E_{q_{2}^{*}(Z;\alpha^{\left(k\right)})}[\log p(Z;\alpha )],$$

where $q_{2}^{*}(Z;\alpha^{\left(k\right)})$ denotes the approximation of q_2_ given current hyperparameter(s) **α** ^(k)^. Here, we use that other terms of both the approximate posterior and the conditional log‐likelihood disappear because they do not contain **α**(as exemplified for the conditional log‐likelihood (20) for the spike‐and‐slab model). Often, (10) can be analytically maximized as in Joo (2017) for the Bayesian Lasso, as implemented in R‐package BLasso.

A general concern with VB approximations is the potential underestimation of posterior variances (Blei et al., 2017). However, for EB estimation of the hyperparameters, the variation across high‐dimensional parameters, which is modeled by the prior with parameter(s) **α**, is deemed more relevant than the posterior variances themselves. This suggests combining VB EB with MCMC: use VB for computational efficiency to iteratively estimate **α**, followed by one MCMC run with fixed **α** to obtain more accurate posteriors. For the latter, the VB posterior mode estimates provide a warm start for the sampling. Because of the connection between VB and Gibbs sampling (Gelfand & Smith, 1990), it is usually fairly straightforward to develop a Gibbs sampler once a VB approximation is available.

### 2.3 Moment EB

An alternative to MML (4) is moment estimation, which is discussed below. In case p (univariate), models share a prior (as discussed above), equating theoretical moments to empirical moments is a textbook example on EB. In prediction, however, we often have only one model. Now, assume that we have an initial estimate $\hat{\theta }=\hat{\theta }(Y)$. Moreover, ${\left(\theta_{j}\right)}_{j=1}^{p}$ share prior π_**α**_, with, say, **α**=(α_1_,α_2_). Then, α_1_ and α_2_ can be estimated by solving moment equations if the conditional moments $E[{\hat{\theta }}_{j}(Y)\;|\;\theta ]$ and $E[{\hat{\theta }}_{j}^{2}(Y)\;|\;\theta ]$ are analytically tractable as functions f_1_ and f_2_ of **θ**:

(11) $$\begin{array}{ll} \frac{1}{p}\sum_{j}{\hat{\theta }}_{j}\approx \frac{1}{p}\sum_{j}E\left[{\hat{\theta }}_{j}(Y)\right] & =\frac{1}{p}\sum_{j}E_{\pi_{\alpha }}\left[E\left[{\hat{\theta }}_{j}(Y)\;|\;\theta \right]\right]=\frac{1}{p}\sum_{j}E_{\pi_{\alpha }}\left[f_{1}(\theta )\right]:=h_{1}(\alpha_{1},\alpha_{2})\\ \frac{1}{p}\sum_{j}{\hat{\theta }}_{j}^{2}\approx \frac{1}{p}\sum_{j}E\left[{\hat{\theta }}_{j}^{2}(Y)\right] & =\frac{1}{p}\sum_{j}E_{\pi_{\alpha }}\left[E\left[{\hat{\theta }}_{j}^{2}(Y)\;|\;\theta \right]\right]=\frac{1}{p}\sum_{j}E_{\pi_{\alpha }}\left[f_{2}(\theta )\right]:=h_{2}(\alpha_{1},\alpha_{2}), \end{array}$$

where h_1_ and h_2_ are known functions. In a group‐regularized logistic ridge regression setting, van de Wiel, Lien, Verlaat, van Wieringen, and Wilting (2016) use a similar idea. Here, groups of variables are given ( $G_{g}$; e.g., gene sets), corresponding to priors θ_j_∼N(0, $\alpha_{g}$) if $j\in G_{g}$. They first use a standard ridge estimator for $\hat{\theta }(Y)$ and then derive and solve G estimating equations with G unknowns to estimate $\alpha ={\left(\alpha_{g}\right)}_{g=1}^{G}$:

(12) $$\frac{1}{p}\sum_{j\in G_{g}}{\hat{\theta }}_{j}^{2}\approx \frac{1}{p}\sum_{j\in G_{g}}E_{\pi_{\alpha }(\theta )}\left[E[{\hat{\theta }}_{j}^{2}(Y)\;|\;\theta ]\right]=\frac{1}{p}\sum_{j\in G_{g}}E_{\pi_{\alpha }}\left[f_{g}(\theta )\right]:=h_{g}(\alpha )\;\forall g=1,\text{…},G.$$

Le Cessie and van Houwelingen (1992) provide expressions for the mean and variance of the logistic ridge estimator, rendering $f_{g}(\theta )=E[{\hat{\theta }}_{j}^{2}(Y)\;|\;\theta ]={\left(E[{\hat{\theta }}_{j}(Y)\;|\;\theta ]\right)}^{2}+V[{\hat{\theta }}_{j}^{2}(Y)\;|\;\theta ]$. Because of the bias introduced by penalization, the mean term and hence $f_{g}$ depends on all θ_j_'s (not just those for which $j\in G_{g}$), so $h_{g}$ depends on all $\alpha_{g}$'s. This leads to a system of G linear equations with G unknowns. For several cancer genomics applications, van de Wiel et al. (2016) and Novianti, Snoek, Wilting, and van de Wiel (2017) show that using group penalty parameters that are inverse proportional to solution ${\hat{\alpha }}_{g}$ improves predictive performance.

Note that the comparison between likelihood‐based (Section 2.2) and moment‐based estimation is on a somewhat different footing here than for ordinary parameter estimation. In the latter case, likelihood‐based estimation is usually preferred because the estimator has several optimality properties when the likelihood is correctly specified. For many types of data and models, the appropriateness of the likelihood can be verified with a variety of techniques. The latter, however, is much harder for the prior, which contains the hyperparameters. The moment estimator depends less on the parametric form of the prior than the marginal likelihood‐based one, so it may be more robust against misspecification of the prior.

## 3 EB AND CROSS‐VALIDATION FOR MULTIPLE HYPERPARAMETERS

Cross‐validation (CV) is a powerful, alternative principle to obtain hyperparameters, usually referred to as tuning parameters in this context. A practical asset of CV is that it is easy to implement when the number of tuning parameters is low. Moreover, it allows to directly optimize the tuning parameter with respect to the out‐of‐bag predictive performance, thereby matching directly with the main goal of most prediction problems. However, CV can be computationally unattractive when a) model fitting takes considerable time, like for most MCMC‐based solutions, or when b) multiple tuning parameters are required, because the search grid grows exponentially with the number of tuning parameters. In the latter case, sequential tuning approaches could alleviate the computational burden, but because of local optima of the utility function, these may be far from globally optimal as shown for the elastic net (Waldron et al., 2011).

**Figure 1:**
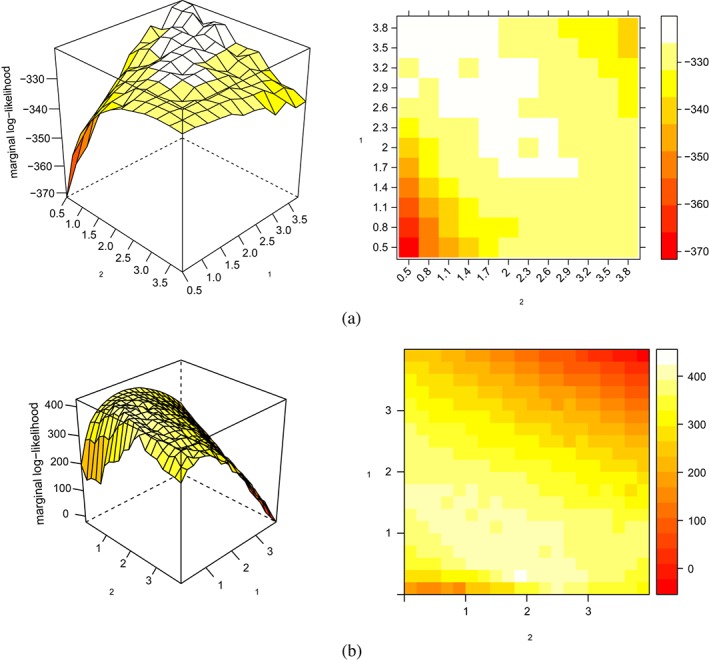
Marginal likelihood (up to constant) as a function of λ_1_ and λ_2_ in the Bayesian elastic net. (a) p=n=200. (b) p=1000,n=100 [Colour figure can be viewed at wileyonlinelibrary.com]

When hyperparameters are “competitive”, for example, when they shrink the same parameters, EB approaches may, like CV, struggle to find the optimal ones because of a flat or multimodal marginal likelihood (4). Figure 1 shows this for the Bayesian elastic net (Li & Lin, 2010). This figure is obtained by estimating the marginal likelihood for varying values of the two hyperparameters in the elastic net. The model and estimation procedure are given in the Supporting Information. The data was simulated by first sampling **X** with independent entries: X_i j_∼N(0,1),i=1,…,n=200, j=1,…,p=200. Next, we generated model parameters β_j_ for j=1,…200 from the elastic net prior with λ_1_,λ_2_=2 and set the response Y_i_=**X**_i_ **β**+ϵ_i_, with ϵ_i_∼N(0,1). A Gibbs sampler was run for every combination of λ_1_,λ_2_∈{0.5,0.8,1.1,…,3.8}, and the marginal likelihood was calculated for every combination. Figure 1a shows that the marginal likelihood estimation indeed renders a high value for the true (λ_1_,λ_2_) combination, (2,2), but many other combinations of one higher and one lower penalty render very similar values. Figure 1b shows that, when we extend the simulation to n=100,p=1000, the marginal likelihood is less flat likely because of the larger p. However, while the true value, (2,2), still corresponds to a high marginal likelihood, a bias towards a smaller L1 penalty is observed.

Practical solutions for the competition between hyperparameters depend on the data, the classifier, and the EB approach employed. For prediction, local optima are not necessarily a problem: the corresponding models likely predict almost equally well. If one desires a sparse solution, one could consider a grid for the sparsity parameter and employ EB to find the other parameter(s) conditional on the sparsity one. Then, one may opt for the smallest model within a prespecified margin of the best performing model, in terms of marginal likelihood or any other criterion. Alternatively, one fixes the a priori expected (or desired) number of included variables, which is feasible for spike‐and‐slab models, and uses EB for other parameters.

Sometimes, it may be worthwhile to combine EB with CV. For example, if one wishes to apply different penalties $\lambda_{g}$ for groups of variables (Boulesteix, De Bin, Jiang, & Fuchs, 2017; van de Wiel et al., 2016), one may reparameterize $\lambda_{g}=\lambda \lambda_{g}^{\prime }$ and optimize the global parameter *λ* by CV with respect to predictive performance while estimating the multipliers $\lambda_{g}^{\prime }$ by EB. Alternatively, CV or similar out‐of‐bag approaches may be used to tune the initial EB estimates to improve predictive performance or to implement parameter thresholding.

## 4 CRITICISMS AND THEORY ON EB

Empirical Bayes comes with assumptions and hence with criticism. Of course, such criticism should be balanced against potential assets of EB, such as computational efficiency and its ability to account for prior information to improve predictions. We discuss three major criticisms and cast these in the high‐dimensional perspective. First, uncertainty of the hyperparameter ***α*** is not propagated as it would be for a fully Bayesian approach. In a high‐dimensional setting, the prior parameters are estimated from a large number of variables. Hence, depending on the correlation strength, the uncertainty may be relatively small. In fact, in a regression variable selection context, Scott and Berger (2010) argue that the uncertainty of the selected model is potentially a larger problem, because the marginal likelihood maximization EB may lead to a degenerate solution, which may be undesirable when alternative values of the hyperparameter(s) render marginal likelihoods that are very close to the optimal one. A hybrid FB–EB approach, as discussed in Section 6.2, may provide the best of both worlds. A second criticism is that EB accommodates the “average ones” not the (possibly more interesting) extremes. In many high‐dimensional applications, however, the use of more complex priors is feasible, for example, mixtures or heavy‐tailed ones. Such priors can better accommodate nonaverage behavior.

A third criticism is the lack of theoretical guarantees on EB, particularly on how and whether an EB estimator improves when *p*(instead of *n*) increases. This is likely because of the complex dependency of the hyperparameters on all variables. In addition, the complex (algorithmic) construction of some EB estimators hampers analytical analysis of their properties. Most theoretical results are available for the simple normal means model (so *p*=*n* and **X**=*I*_*p*_), which allows a factorization of the likelihood as in (3). For example, Johnstone and Silverman (2004) provide asymptotic optimality results for a spike‐and‐slab–type prior. In addition, Belitser and Nurushev (2015) present theoretical evidence that, in a sparse spike‐and‐slab setting, EB allows the use of a Gaussian slab to obtain good contraction rates of the posteriors, which is a prerequisite for obtaining correct coverage of credibility intervals. Such a Gaussian slab prior is not recommended for the ordinary sparse Bayes setting because it shows suboptimal contraction rates as compared with more heavy‐tailed slab distributions (Castillo & van der Vaart, 2012). For a wider class of models, Rousseau and Szabo (2017) recently showed that full (hierarchical) Bayes and MML EB have the same oracle posterior contraction rates (*n*→*∞*) under weak conditions on the hyperprior. Below, we provide some analytical results for an EB estimator of the prior variance of linear regression parameters. Even for this fairly simple model, calculations are fairly tedious.

## 5 EXPECTED MSE FOR A SIMPLE EB ESTIMATOR

We study a very simple EB estimator for linear (ridge) regression to gain insight on how the quality of the estimator, as quantified by the expected mean squared error (EMSE), changes with *p*. We start with the case *p*<*n*, which allows analytical results. This includes the “medium‐dimensional” case with *p* relatively close to *n*, for which regularization is often desirable. Then, the results for *p*≥*n* are obtained by simulation.

### 5.1 Setting 1: Initial ordinary least squares (OLS) estimator

Suppose that *β*_*j*_∼^iid^ *N*(0,*τ* ^2^). Let $\hat{\beta }$ be the OLS estimator of ***β*** in a linear regression model without intercept. For the sake of simplicity, we assume the error variance *σ* ^2^=1 to be known. Then,

$$(\hat{\beta }|X,\beta )∼N(\beta ,V),$$

with *V*=(**X** ^*T*^ **X**)^−1^ and *v*_*j*_=*V*_*j* *j*_. Hoerl, Kennard, and Baldwin (1975) propose the following simple estimator of *τ* ^2^ for *p*<*n*:

(13) $${\left(\tau^{\prime }\right)}^{2}=\frac{\sum_{j=1}^{p}{\hat{\beta }}_{j}^{2}}{p}.$$

Since $E_{Y}({\hat{\beta }}_{j})=\beta_{j}$ and $V_{Y}({\hat{\beta }}_{j})=v_{j}$, we have

$$E_{\beta }(E_{Y}[{\left(\tau^{\prime }\right)}^{2}])=\frac{\sum_{j=1}^{p}\left(v_{j}+E_{\beta }\left(\beta_{j}^{2}\right)\right)}{p}=\frac{\sum_{j=1}^{p}v_{j}}{p}+\tau^{2}.$$

Hence, the estimator can be corrected for this expected bias without inflating the variance:

(14) $${\hat{\tau }}^{2}=\frac{\sum_{j=1}^{p}\left({\hat{\beta }}_{j}^{2}-v_{j}\right)}{p}.$$

We wish to study the properties of ${\hat{\tau }}^{2}$ in terms of *p* and *n*. For that, we consider the EMSE, where the mean squared error is computed w.r.t. **Y**, which is then averaged over samples of both ***β*** (drawn from the Gaussian prior) and **X**. While the latter is often considered as fixed, it is more realistic to assume it random, particularly when **X** denotes (genomic) measurements. This also allows to establish the quality of the estimator across instances of **X**. We assume that, after standardization, *X*_*i*_∼*N*(0,Σ=Σ_*p*×*p*_),*i*=1,…,*n*, with Σ_*j* *j*_=1. Then, we study

(15) $$\text{EMSE}({\hat{\tau }}^{2})=E_{X}\left[E_{\beta }\left[{\text{MSE}}_{Y}({\hat{\tau }}^{2}\;|\;\beta ,X)\right]\right]=E_{X}\left[E_{\beta }\left[{\left(E_{Y}({\hat{\tau }}^{2}\;|\;\beta ,X)-\tau^{2}\right)}^{2}+V_{Y}({\hat{\tau }}^{2}\;|\;\beta ,X)\right]\right].$$

Theorem 1 Let $\text{EMSE}({\hat{\tau }}^{2})$ be as in (15). Then, we have, with Ψ=Σ^−1^, for *p*<*n*−3,

(16) $$\begin{array}{ll} \text{EMSE}({\hat{\tau }}^{2}) & =\frac{2}{(n-p-1)p^{2}}\left[\sum_{j=1}^{p}\left(\frac{2\psi_{jj}^{2}}{\left(n-p-1)(n-p-3\right)}+\frac{\psi_{jj}^{2}}{\left(n-p-1\right)}\right)+2\tau^{2}\sum_{j=1}^{p}\psi_{jj}\right.\\ & \;\left.+\sum_{j,k\neq j}^{p}\left(\frac{(n-p+1)\psi_{jk}^{2}+(n-p-1)\psi_{jj}\psi_{kk}}{\left(n-p)(n-p-1)(n-p-3\right)}+\frac{\psi_{jk}^{2}}{\left(n-p-1\right)}\right)\right]+\frac{2\tau^{4}}{p}. \end{array}$$

See Supporting Information.

Corollary 1 Let ${\text{EMSE}}_{⊥}({\hat{\tau }}^{2})$ be $\text{EMSE}({\hat{\tau }}^{2})$ for independent *X*_*i*_: *ψ*_*j* *j*_=1 and *ψ*_*jk*_=0. Then, for *p*<*n*−3,

(17) $$\begin{array}{ll} {\text{EMSE}}_{⊥}({\hat{\tau }}^{2}) & =\frac{2}{(n-p-1)p}\left[\frac{2}{\left(n-p-1)(n-p-3\right)}+\frac{1}{n-p-1}+2\tau^{2}\right.\\ & \left.+\frac{p-1}{\left(n-p)(n-p-3\right)}\right]+\frac{2\tau^{4}}{p}. \end{array}$$

**Figure 2:**
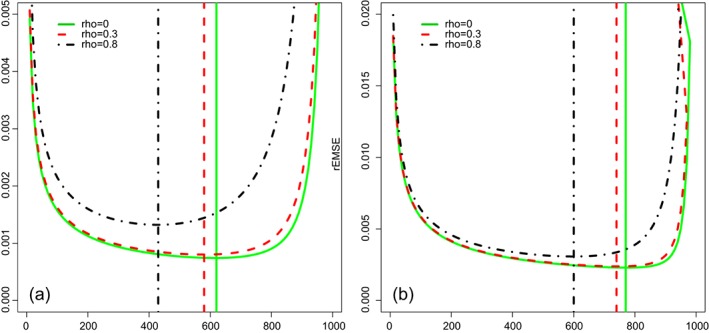
Y‐axis: root expected mean squared error ((16), (17)), X‐axis: p. Settings: n=1000; independent **X**(ρ=0), ρ=0.3,0.8; b=10. (a) τ ^2^=0.01. (b) τ ^2^=0.04. Vertical line denotes the minimum [Colour figure can be viewed at wileyonlinelibrary.com]

Equations  (16) and (17) clearly show the balance for increasing *p*, causing *n*−*p* to decrease. From (16), we observe that the effect of collinearity in **X** may be large when the number of nonzero *ψ*_*jk*_'s (i.e., partial correlations) is large because of the double summation and the relatively small O(n−p) denominator of $\psi_{jk}^{2}$. In addition, we observe that, for large *τ* ^2^, a large *p* is relatively more beneficial than for small *τ* ^2^. Figure 2 shows the root EMSE as function of *p*<*n* for *n*=1000 for *τ* ^2^=(0.1)^2^=0.01;*τ* ^2^=(0.2)^2^=0.04 for Σ=*I*_*p*_ (referred to as “independent **X**”); $\Sigma =I_{B}⊗A_{b\times b}^{\rho }$, (block‐correlation) with *b* as the block size and *B*=*p*/*b* as the number of blocks, $A_{jj}^{\rho }=1,A_{ij}^{\rho }=\rho$, where *ρ* denotes the correlation between any two variables *i*≠*j*. We show results for *b*=10 and *ρ*=0.3,0.8; results for *b*=50 were fairly similar. The figures support the conclusions drawn from studying the equations.

### 5.2 Setting 2: Initial ridge estimator

**Figure 3:**
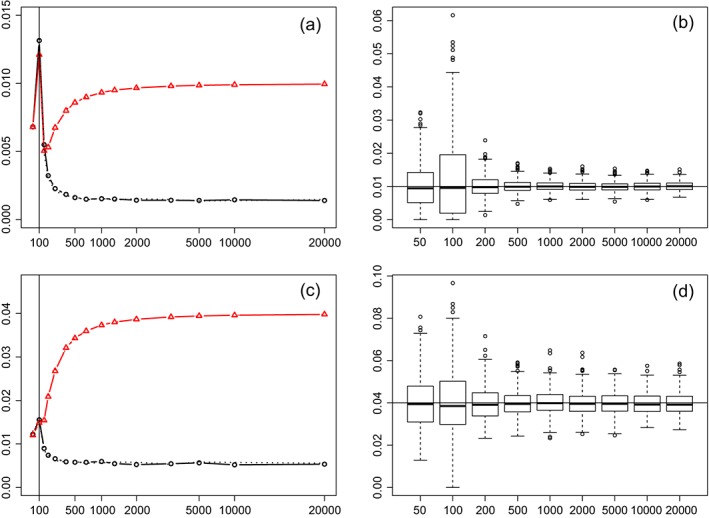
Subfigures (a) and (c): root EMSE (y‐axis) versus p (x‐axis; square‐root scale) for τ ^2^=0.01,0.04 and n=100. Red triangles: estimator ${\hat{\tau }}^{2}$ (14), black circles: bias‐adjusted estimator ${\hat{\tau }}_{2}^{2}$ (18). Solid lines: independent **X**, dotted line: block‐correlation, b=50,ρ=0.8. Vertical line denotes p=n=100. Subfigures (b) and (d): corresponding box‐plots of ${\hat{\tau }}_{2}^{2}$ for 500 simulations in the independent **X** setting [Colour figure can be viewed at wileyonlinelibrary.com]

It is not straightforward to extend the formulas above to the penalized *p*>*n* setting because i) penalization introduces bias in the estimates, so $E_{Y}({\hat{\beta }}_{j}^{\lambda })\neq \beta_{j}$; ii) unlike (*X* ^*T*^ *X*)^−1^, (*X* ^*T*^ *X*+*λ* *I*_*p*_)^−1^ does not follow an inverse‐Wishart distribution. Hence, we approximate the EMSE by simulations. In the penalized setting, estimators of *τ* ^2^ more advanced than (14) are available (Cule & De Iorio, 2013). We proposed an alternative that accounts for the bias of ${\hat{\beta }}_{j}^{\lambda }$ because of penalization (van de Wiel et al., 2016):

(18) $${\hat{\tau }}_{2}^{2}=\frac{\sum_{j=1}^{p}\left({\left({\hat{\beta }}_{j}^{\lambda_{0}}\right)}^{2}/v_{j}-1\right)}{\sum_{j,k=1}^{p}v_{j}^{-1}c_{jk}^{2}},$$

where *c*_*jk*_ is the known coefficient of the bias $E_{Y}({\hat{\beta }}_{j}^{\lambda })=\sum_{k=1}^{p}c_{jk}\beta_{k}$ (van de Wiel et al., 2016), and *λ*_0_ is an initial value of *λ*. We used *λ*_0_=1, corresponding to a fairly noninformative initial $N(0,\tau_{0}^{2}=1)$ prior for *β*_*j*_. Results were rather insensitive to the exact value of *λ*_0_. Figure 3 shows the root EMSE, estimated from 500 generations of **X**, ***β***, and **Y** per setting, using the settings as above except for *n*=100, where *p*≤20,000 and *b*=50.

**Figure 4:**
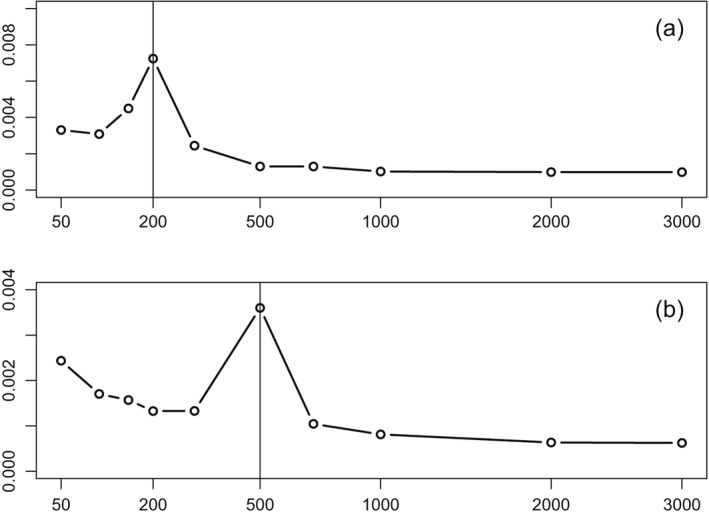
Y‐axis: root expected mean squared error of ${\hat{\tau }}_{2}^{2}$ (18); X‐axis: p (square‐root scale). Settings: τ ^2^=0.01; independent **X**. (a) n=200. (b) n=500. Vertical line denotes p=n. Results are based on 200 simulations

From Figure 3, we observe that ${\hat{\tau }}^{2}$ (14) and ${\hat{\tau }}_{2}^{2}$ (18) are competitive for *p*≤*n*, but the bias‐corrected estimator ${\hat{\tau }}_{2}^{2}$ is far superior for *p*≫*n*. In fact, the latter is very well on target for *p*≥500, supporting the notion that large *p* is beneficial for EB. Interestingly, even fairly strong correlation seemed to have little impact on the performance (the dotted lines largely overlap the solid ones). This is possibly due to the de‐correlation effect of the initial ridge regression with penalty *λ*_0_=1. This small penalty (much smaller than the true values *λ*_true_=1/*τ* ^2^=1/0.01=100;1/0.04=25) seems to suffice to initially regularize *X* ^*T*^ *X*, which explains why the performance improves after *p*=*n*. A striking aspect is that across the range of *p*, the performance of ${\hat{\tau }}_{2}^{2}$ is the worst for *p*≈*n*=100. A smaller simulation for *n*=200,500 shows the same phenomenon visible from Figure 4. In addition, the use of an even vaguer Gaussian prior with $\tau_{0}^{2}=10\Rightarrow \lambda_{0}=0.1$ leads to a similar and even somewhat more pronounced pattern in terms of the peak of root EMSE around *p*=*n* (data not shown). An explanation is that, for *p*<*n*, the estimation of ***β*** is stable and well conditioned, while the fairly weak penalty introduces little bias. Thus, even though *p* is small, the information from each ${\hat{\beta }}_{j}^{\lambda_{0}}$ is solid, which benefits the estimation of *τ* ^2^. For *p*≈*n*, the penalty necessarily introduces a larger bias in the estimation of *β*_*j*_, whereas the EB estimator does not yet profit much from a large *p* as is the case for *p*>*n*. Others have noted this “peaking around *p*=*n* phenomenon” as well, for example, in the context of test error for (regularized) linear discriminant analysis (Duin, 2000).

Finally, it is tempting to compare the EB estimates of *τ* ^2^ with CV estimates. We noticed that both five‐fold and ten‐fold CV (minimizing cross‐validated mean squared prediction error for given **X**) rendered estimates of *τ* ^2^ with a root EMSE substantially larger than that of ${\hat{\tau }}_{2}^{2}$. For example, for *τ* ^2^=0.01,*p*=1000,*n*=100, and independent *X*_*i*_(so Σ=*I*_*p*_), $\text{root EMSE}({\hat{\tau }}_{\text{CV10}}^{2})=0.0064$, whereas $\text{root EMSE}({\hat{\tau }}_{2}^{2})=0.0015$. However, one should bear in mind that CV aims at minimizing prediction error rather than at estimating *τ* ^2^. In fact, we noticed that the predictive performances usually differed very little when using either $\lambda_{\text{CV10}}={\hat{\tau }}_{\text{CV10}}^{-2}$ or $\lambda_{\text{EB}}={\hat{\tau }}_{2}^{-2}$. Nevertheless, a practical advantage of the EB estimate is its computational efficiency (Cule & De Iorio, 2013): It requires only one ridge‐fit, whereas *k*‐fold CV requires *k* times the number of ridge‐fits per fold (which depends on the efficiency of the search and the use of approximations).

## 6 APPLICATION OF EB WHEN USING CO‐DATA

Tai and Pan (2007) and Novianti et al. (2017) present specific data examples on how co‐data can help improve prediction and variable selection in high‐dimensional setting. Here, we present two novel prediction examples, both of which use EB to account for co‐data.

### 6.1 MCMC EB for spike‐and‐slab models

Consider a high‐dimensional generalized linear model setting, where response **Y** is linked to **X** via the linear predictor *η*=**X** ***β***. Moreover, *β*_*j*_ is endowed with a spike‐and‐slab prior of the form

$$(\beta_{j}\;|\;\xi_{j}=0)∼F_{0},\;(\beta_{j}\;|\;\xi_{j}=1)∼F_{1},\;\xi_{j}∼\text{Bern}(\nu_{j}),\;j=1,\text{…},p,$$

where, typically, *F*_0_ is concentrated around zero or even *F*_0_=*δ*_0_, and *F*_1_ is more dispersed, for example, Gaussian (Newcombe et al., 2014) or Laplace (Ročková & George, 2014). The alternative mixture prior representation is obtained by marginalization over the latent variables *ξ*_*j*_. The model may contain additional nuisance parameters that do not depend on *ξ*_*j*_ (such as error variance *σ*), which we omit in the notation below. Now, assume that we have (several) additional sources of information on the *p* variables coded by a *p*×*s* co‐data matrix *C* with *s*≪*p*. Let us model the prior inclusion probability *ν*_*j*_ parsimoniously as a function of the co‐data

(19) $$\nu_{j,\alpha }=g^{-1}(C_{j}\alpha ),$$

where *C*_*j*_ is the *j*th row of *C*, ***α*** is an *s*×1 vector of hyperparameters, and g is a link function, for example, a logit link. The EB task is to estimate the hyperparameters ***α***. Suppose that we have an MCMC sampler that renders posterior samples for all parameters, including the latent ones, given current hyperparameters ***α*** ^(*k*)^. Then, the conditional log‐likelihood in (9) is equal to

(20) $$\begin{array}{ll} \ell (Y,\theta^{m,(k)};\alpha ) & =\log\pi (Y,\beta^{m,(k)},\xi^{m,(k)};\alpha )\\ & =\log\pi (Y\;|\;\beta^{m,(k)})+\log\pi (\beta^{m,(k)}\;|\;\xi^{m,(k)})+\log\pi (\xi^{m,(k)};\alpha ). \end{array}$$

Hence, only the last term depends on ***α***, so (9) reduces to finding

$$\begin{array}{ll} {\text{argmax}}_{\alpha } & \left\{\sum_{m=1}^{M}\sum_{j=1}^{p}\log\left[\text{Bern}\left(\xi_{j}^{m,(k)};\nu_{j,\alpha }\right)\right]\right\}\\ & ={\text{argmax}}_{\alpha }\left\{\sum_{j=1}^{p}\sum_{m=1}^{M}\log\left[\text{Bern}\left(\xi_{j}^{m,(k)}\right);\left.\nu_{j,\alpha }\right)\right]\right\}\\ & ={\text{argmax}}_{\alpha }\left\{\sum_{j=1}^{p}\log\left[\text{Bin}\left(\sum_{m=1}^{M}\xi_{j}^{m,(k)}\right);\left.M,\nu_{j,\alpha }\right)\right]\right\}. \end{array}$$

The latter equality holds because *ν*_*j*,***α***_ does not depend on *m*=1,…,*M* and the Bin(*M*,*q*) density differs from the product of *M* Bern(*q*) densities only by a binomial factor that does not depend on ***α***. Hence, estimating ***α*** reduces to binomial regression of “observations” $B_{j}^{k}=\sum_{m}\xi_{j}^{m,(k)},j=1,\text{…},p$, on the *s* columns of design matrix *C*. The previous estimate is then iteratively updated by this one as in (8) for a new round of MCMC sampling.

The reduction to simple regression is feasible because of the factorization (20) and the i.i.d. Bernoulli prior for *ξ*_*j*_. Other Bayesian sparse regression models like the Bayesian elastic net (see Section 3) can also be represented as (scale) mixtures but with a remaining dependency of ***β*** on ***α*** plus a more complex dependency of the mixture proportions on ***α***. While conceptually simple, the algorithm above is computationally demanding, requiring efficient implementations of spike‐and‐slab MCMC (such as those by Peltola, Marttinen, & Vehtari, 2012, and Newcombe et al., 2014). Variational Bayes approximations may be an alternative (Carbonetto & Stephens, 2012) in combination with an EM‐type maximization (Beal & Ghahramani, 2003).

### 6.2 Simulation example: Interval estimation

#### 6.2.1 Empirical Bayes versus full Bayes

Empirical Bayes is not “truly” Bayes because the prior parameters are fixed after estimating these from the data. A disadvantage of many full Bayes settings, however, is the computational time: The extra layer of priors may lead to a strong increase, for example, from seconds to minutes (see the example of Bar and Schifano (2011) with 2,000 variables) or from minutes to several hours. For the multivariate low‐dimensional setting, Carlin and Louis (2000) show that, despite their lack of error propagation, EB methods can be rather competitive to full Bayes ones in terms of frequentist coverage probabilities of the parameter credible intervals. Below, we compare Bayes, EB, and hybrid credible intervals for predictions in medium‐dimensional settings with *p* of the same order of magnitude as *n*.

#### 6.2.2 Setting

As indicated in the introduction, the medium‐dimensional case is likely to become more and more relevant in clinical prediction. In a clinical setting, the uncertainty of each individual's prediction is of importance. The Bayesian paradigm lends itself well for obtaining interval estimates in (penalized) regression settings because it allows the uncertainty propagation of the tuning parameter(s). In the low‐dimensional Bayesian linear regression setting, Morris (1983) and Basu, Ghosh, and Mukerjee (2003) provide theoretical guarantees for the coverage of an EB interval, which accounts for the uncertainty of the prediction and the shrinkage factor. In a Bayesian logistic regression setting, we compare three models for the priors of the coefficients *β*_*j*_ in terms of coverage of the posterior predictive intervals. These models differ in the level of error propagation. We assume that the variables are grouped into *G* groups on the basis of co‐data (Tai & Pan, 2007; van de Wiel et al., 2016).

#### 6.2.3 Models

Denote the groups of variables by $G_{g},g=1,\text{…},G$. We assume that

(21) $$\begin{array}{ll} Y_{i} & ∼\text{Bernoulli}\;(\text{expit}(X_{i}\beta ))\\ \beta_{j} & ∼N\left(0,\tau_{g}^{2}\right),j\in G_{g}, \end{array}$$

where expit(x)=exp(x)/(1+exp(x)). We consider three models for precisions $\tau_{g}^{-2}$. First, the EB model

(22) $$\tau_{g}^{-2}=\lambda \lambda_{g}^{2},$$

where *λ* and $\lambda_{g}$ are fixed. Second, the (conjugate) FB model

(23) $$\tau_{g}^{-2}∼\Gamma (\alpha_{1},\alpha_{2}),$$

with *α*_1_ and *α*_2_ such that the prior is rendered uninformative. Third, the hybrid model

(24) $$\begin{array}{ll} \tau_{g}^{-2} & =\tau^{-2}\lambda_{g}^{2}\\ \tau^{-2} & ∼\Gamma (\alpha_{1},\alpha_{2}), \end{array}$$

with *α*_1_ and *α*_2_ such that the prior is rendered uninformative and $\lambda_{g}$ fixed.

Model (22) is equivalent to the one used in (van de Wiel et al., 2016). We estimate the global ridge tuning parameter *λ* by cross‐validation and the group multipliers $\lambda_{g}$ by moment‐based EB as in (12). This model generally renders good point predictions and is computationally very efficient. It may, however, not suffice for interval estimation because the uncertainty of ${\hat{\tau }}_{g}^{-2}$ is not accounted for. Model (23) renders a classical Bayesian random effects model. It may be the preferred model when *G* is small and the number of features per group is large: the estimation of $\tau_{g}^{-2}$ will be relatively precise, and the uncertainty of *τ* is propagated. However, this model is computationally cumbersome for large *G* because of the large number of hyperpriors, which need to be integrated out when computing the posterior of ***β***. Moreover, when some groups are small, the imprecise estimation of $\tau_{g}^{-2}$ may render inferior predictions. Model (24) is a compromise: It contains only one random hyperparameter, *τ*. Thus, model (24) is computationally efficient while still propagating uncertainty of *τ*. We assume the group‐specific penalty multipliers $\lambda_{g}$ to be identical to those in model (22) to ensure comparability.

#### 6.2.4 A small simulation

In combination with (21), (22) to (24) render three Bayesian models that are implemented using the R‐package INLA (Rue, Martino, & Chopin, 2009) after substituting the estimated *λ* and $\lambda_{g}$'s into model (22) and $\lambda_{g}$ into (24). We evaluate 95% posterior intervals for the prediction probabilities on an event *q*_*i*_=expit(**X**_*i*_ ***β***). We consider equal‐tailed intervals and highest probability density (HPD) intervals (Carlin & Louis, 2000). The latter concentrates more around the posterior mode, so it may be less vulnerable to shrinkage than the first. The following simulation settings were used for model (21):

- # groups *G*=2,5. # variables per group: *p*_*G*_. Total *#* variables: *p*=*p*_*G*_∗*G*.
- For *G*=2, all 3 models are applied; *n*_train_=*n*_test_=100,*p*_*G*_=10,20,30,40,50.
- For *G*=5, models (22) and (24) are applied; *n*_train_=200,*n*_test_=100,*p*_*G*_=10,20,40. Model (23) was not evaluated for this computationally demanding case.
- For variable *j* in group g=1,…,G, $\beta_{j}∼N(0,\tau_{g}^{2})$, where $\tau_{g}=\tau_{0}2^{-(g-1)}$, so prior standard deviations decrease by a factor of 2 for each next group g; *τ*_0_ is calibrated such that ≈20*%* of observations render extreme probabilities *q*_*i*_(<0.05 or >0.95).
- Correlation between variables occurs in blocks of 5, with correlation *ρ*=0.1.
- Each simulation setting was repeated *n*_rep_=50 times; coverage of 95% posterior intervals for *q*_*i*_,*i*=1,…,*n*_test_, is studied.

We also considered *ρ*=0.5 and constant *β*'s within each group (hence not obeying the Gaussian prior). Results were very similar and hence not shown.

#### 6.2.5 Results

**Figure 5:**
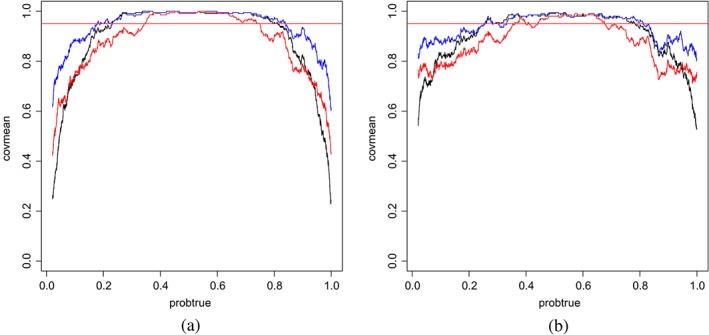
X‐axis: True event probability q_j_; Y‐axis: mean coverage of 95% posterior intervals for event probability. Mean is estimated by moving average. Case: $G=2,p_{g}=30,p=G*p_{G}=60,n_{\text{train}}=100$. Methods: hybrid (blue), empirical Bayes (black), full Bayes (red). (a) Equal‐tailed interval; (b) HPD interval [Colour figure can be viewed at wileyonlinelibrary.com]

**Figure 6:**
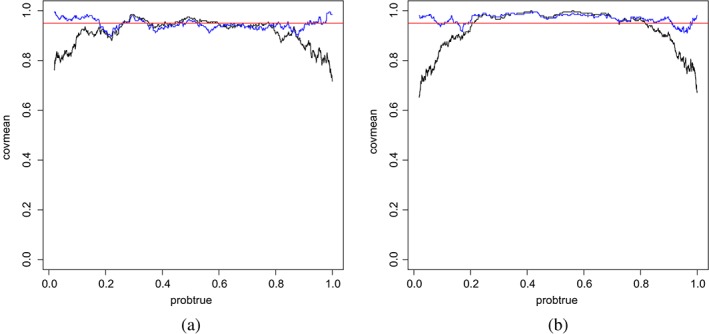
X‐axis: True event probability q_j_; Y‐axis: mean coverage of 95% HPD intervals for event probability. Mean is estimated by moving average. Cases: $G=5,p_{g}=10,40;p=G*p_{G}=50,200;n_{\text{train}}=200$. Methods: hybrid (blue), empirical Bayes (black). (a) $p_{g}=10,G=5$. (b) $p_{g}=40,G=5$ [Colour figure can be viewed at wileyonlinelibrary.com]

We focus on the intervals here; the results on the point predictions (posterior modes) of *q*_*i*_ are rather similar for models (22) to (24). We compute the average coverage of the true *q*_*i*_ by the 95% intervals across all test samples, averaged over *n*_rep_ repeats. We then plot *q*_*i*_ versus the moving average coverage on overlapping windows of 200 predictions. These are displayed in Figures 5 and 6 for two simulation settings; see the Supporting Information for other settings. First, from Figure 5, it is clear that HPD intervals outperform their equal‐tailed counterparts, particularly for the extreme *q*_*i*_'s. Equal‐tailed intervals are more sensitive to the bias introduced by penalization, which is stronger for the extremes. This is in line with the findings of (Carlin & Louis, 2000). Possibly more surprising is the somewhat inferior coverage for the FB model (23) in this simulation. It may result from use of the conjugate, but possibly wrong prior in (23), or from the small value of *p*_*G*_. The counterpart, the EB model, performs better but still renders too low coverage for the extremes. This likely results from too narrow intervals caused by lack of propagation of the uncertainty of the global penalization parameter, *τ* ^−2^∝*λ*. The hybrid model (24) seems to correctly balance the empirical Bayes estimation of the groupwise parameters and the full Bayes handling of *τ* ^−2^. Because of shrinkage, a small bias for the coverage remains for extreme *q*_*i*_'s.

## 7 DISCUSSION AND EXTENSIONS

We showed that EB is a versatile and powerful approach to “learn from a lot” in two ways: first, from the large number of variables and, second, from prior information on the variables, stored as co‐data. We reviewed several methods for EB estimation in a broad spectrum of prediction frameworks. This illustrated that developing EB estimators of penalty, prior or other tuning parameters, ranges from simple to challenging, depending on the prediction framework and the ambition in terms of number of hyperparameters to estimate. While EB can be regarded as a “competitor” for cross‐validation and FB in a frequentist or Bayesian setting, respectively, we argued that hybrid solutions may prove useful to exploit the strengths of the approaches.

In the Bayesian framework, maximization of the marginal likelihood is the default EB criterion. This is often computationally intensive. Variational Bayes, which returns a lower bound for the marginal likelihood, in combination with EM‐type optimization, can strongly alleviate the computational burden (Beal & Ghahramani, 2003). It requires careful development of the approximations for the model at hand and verification of accuracy (e.g. by Gibbs sampling) for numerical examples. Alternatively, in a variable selection setting, one may settle for a conditional EB approach (George & Foster, 2000) by conditioning on the included variables, thereby avoiding integration over the large model space.

This overview is by no means complete. Specific EB methods have been developed, particularly also for model‐free predictors. For example, for the random forest, Taddy, Chen, and Yun (2015) estimate the trunk of the trees, which may stabilize results and save considerable computing time compared to a fully Bayesian approach. Te Beest, Mes, Wilting, Brakenhoff, and van de Wiel (2017) demonstrate that co‐data may be used to improve random forest predictions by moderating the sampling weights of the variables.

As illustrated, EB allows to account for co‐data but is not the only way. Full Bayes alternatives exist, particularly for the purpose of variable selection (Ishwaran & Rao, 2005; O'Hara & Sillanpää, 2009; Quintana & Conti, 2013). These are generally computationally very demanding for typical high‐dimensional settings with a large number of variables. Moreover, frequentist solutions have been proposed, which usually require additional tuning parameter(s) to cross‐validate (Bergersen, Glad, & Lyng, 2011; Jiang, He, & Zhang, 2016b) or a group penalty (Meier, van de Geer, & Bühlmann, 2008; Simon, Friedman, Hastie, & Tibshirani, 2013). The latter may perform less well than EB‐based regularization per group when the number of groups is small (Novianti et al., 2017). However, a group penalty may be particulary powerful when the number of groups is large given its much more parsimonious representation of the group structure. Combination of the two principles is an interesting future research direction.

“Empirical Bayes is still in its adolescence” (Efron, 2010), which is particularly true for high‐dimensional prediction and variable selection. More theory on the quality of the estimators as a function of *n* and *p* for a variety of prediction models will be welcomed by the community. Moreover, EB theory for large *p* settings is an active field of research, which will likely lead to more general results. From a practical perspective, prediction accuracy can always be estimated by (repetitive) training/test splits, which allows the evaluation of the EB prediction versus alternatives for the data at hand. The evaluation of variable selection is more difficult. We find it useful to compare indirectly by evaluating the predictive accuracies of models of the same size. This enabled us to show that co‐data‐based EB may improve the predictive performance of small models (Novianti et al., 2017). New prediction methods with various types of penalties, priors, or other tuning parameters are frequently introduced. These may benefit from dedicated EB estimators, particularly when multiple tuning parameters are involved. Extension of EB methods toward estimation of multivariate priors should allow to better accommodate network‐type information (Ročková & George, 2014; Stingo, Chen, Tadesse, & Vannucci, 2011). Finally, priors that are modeled as a function of various sources of co‐data are increasingly relevant in this “Big Data era”. Developing EB estimators of hyperparameters of such priors will require either a parsimonious representation or regularization on the level of hyperparameters to avoid overfitting.

## Supporting information



SJOS_12335‐sup‐0001‐SupplInfEBgenome.pdf
