# Supplementary material for: Learning from a lot: Empirical Bayes for high‐dimensional model‐based prediction
Source: Scand Stat Theory Appl. 2018 Jun 1;46(1):2–25. doi: 10.1111/sjos.12335 (PMC6472625; doi:10.1111/sjos.12335)
Supplement: Supplementary file 1 — SJOS_12335‐sup‐0001‐SupplInfEBgenome.pdf [file SJOS-46-2-s001.pdf]

Supporting Information to "Learning from a lot: Empirical Bayes for high-dimensional model-based prediction"

Mark A. van de Wiel[1,2], Dennis te Beest[1], Magnus Münch[1,3]

1. Dep of Epidemiology and Biostatistics, Amsterdam Public Health research institute, VU University Medical Center, Amsterdam, The Netherlands
2. Dep of Mathematics, VU University, Amsterdam, The Netherlands
3. Mathematical Institute, Faculty of Science, Leiden University, Leiden, The Netherlands

1 Baseball batting example, revisited

We shortly revisit the famous baseball batting example (Efron and Morris, 1975), often used as a scholarly example of EB. While this is an estimation problem instead of a prediction problem, we revisit it for several reasons: i) it is a well-known example for which the true values are known; ii) the EB objective function is the same as for diagonal linear discriminant analysis; and iii) by casting the problem to a large p setting it allows us to show the importance of p being large.

For 18 baseball players, their batting averages over the first 45 bats are recorded and denoted by B_i. The batting averages over the remainder of the season are also known, and considered to be the truth. We follow Van Houwelingen (2014) by modeling $B_i \sim N(\theta_i, \sigma_i^2)$, where the aim is to estimate θ_i. The variances are estimated by $\hat{\sigma}_i^2 = B_i(1-B_i)/45$. Then, to effectuate shrinkage Van Houwelingen (2014) applies a Gaussian prior $N(\mu, \tau^2)$ to θ_i. In the formulation of the marginal likelihood (see Main Document), this implies hyper-parameter $\boldsymbol{\alpha} = (\mu, \tau^2)$, and estimation of $\boldsymbol{\alpha}$ is straightforward due to the conjugacy of the likelihood and the prior: $\hat{\tau}^2 = V(B_i) - \overline{\hat{\sigma}_i^2}$ and $\hat{\mu} = \sum_{i=1}^n w_i B_i / \sum_{i=1}^n w_i$, with $w_i = (\hat{\tau}^2 + \hat{\sigma}_i^2)^{-1}$. Then, the posterior mean estimate equals $\hat{\theta}_i = E(\theta_i | B_i; (\hat{\mu}, \hat{\tau}^2)) = \hat{\mu} + \hat{\tau}^2(\hat{\tau}^2 + \hat{\sigma}_i^2)^{-1}(B_i - \hat{\mu})$. The conclusion in Van Houwelingen (2014) is that the shrinkage prior slightly reduces the mean squared error, but enforces too strong shrinkage for the extremes. E.g. for the best player $\hat{\theta}_1 = 0.271$, whereas $X_1 = 0.400$ and true $\theta_1 = 0.346$. Two possible explanations come to mind: the estimate of the prior parameters is not good due to $p = n$ being small and/or the prior does not accommodate the extremes well. We investigate this.

First, we simulate 10,000 additional true values from a density estimate with Gaussian kernel (using R's `density` command) applied to $(\theta_1, \ldots, \theta_{18})$. To obtain B_i, $i = 19, \ldots, 10018$, Gaussian noise was added with variances $\theta_i(1-\theta_i)/45$. The estimates obtained in Van Houwelingen (2014) were $\hat{\mu} = 0.256$ and $\hat{\tau}^2 = 0.000623$. The latter seems to be a major cause of over-shrinkage: the true variance computed from the 18 known θ_i's equals 0.00143. If we estimate τ^2 from the large data set, a much better estimate is obtained: $\tilde{\tau}^2 = 0.00195$, as compared to the variance of the 18 known plus 10,000 generated true θ_i's, equaling 0.00166. From this, we obtain posterior mean estimate $\tilde{\theta}_1 = 0.293$, which is substantially closer to $\theta_1 = 0.346$ than $\hat{\theta}_1 = 0.271$. Estimates for all 18 players are displayed in Figure 1(a).

In this example, it is natural to replace the Gaussian prior by a 3-component Gaussian mixture prior (bad, mediocre and good players): $\theta_i \sim \sum_{k=1}^3 p_k N(\mu_k, \tau_k^2)$. Then, $\boldsymbol{\alpha}$ consists of 8 hyper-parameters given that $p_3 = 1 - p_1 - p_2$. We employed the EM-type algorithm of Van de Wiel et al. (2012) to maximize the marginal likelihood (see Main Document) in terms of $\boldsymbol{\alpha}$. Here, we

use that the likelihood is Gaussian, and the Gaussian mixture prior is conjugate to it. The latter also facilitates straightforward computation of the shrunken estimator $\hat{\theta}_i^{\text{Mixt}} = E(\theta|B_i; \boldsymbol{\alpha})$. In this setting, the mixture prior is fairly close to the estimated Gaussian prior, and so are the shrunken estimates, as displayed in Figure 1(b). Slightly less shrinkage for the extremes is observed, though. For example, $\hat{\theta}_1^{\text{Mixt}} = 0.298$.

2 Bayesian elastic net

The Bayesian linear elastic net model, as used in the Main Document, is (Li and Lin, 2010):

$$\begin{aligned}
\mathbf{Y}|\mathbf{X}, \boldsymbol{\beta}, \sigma^2 &\sim \text{N}(\mathbf{X}\boldsymbol{\beta}, \sigma^2\mathbf{I}) \\
\boldsymbol{\beta}|\sigma^2 &\sim \prod_{j=1}^{p} g(\lambda_1, \lambda_2, \sigma^2) \cdot \exp\left[-\frac{1}{2\sigma^2}(\lambda_1|\beta_j| + \lambda_2\beta_j^2)\right] \\
\sigma^2 &\sim f(\sigma^2),
\end{aligned}$$

with some arbitrary (possibly improper) density $f(\sigma^2)$. The normalizing constant $g(\lambda_1, \lambda_2, \sigma^2)$ is given by:

$$g(\lambda_1, \lambda_2, \sigma^2) = \sqrt{\frac{\lambda_2}{4\sigma^2}}\phi\left(\frac{\lambda_1}{\sqrt{4\lambda_2\sigma^2}}\right)\Phi\left(\frac{-\lambda_1}{\sqrt{4\lambda_2\sigma^2}}\right)^{-1}$$

Since the simulations are for illustrative purposes only, the error variance was kept fixed at its true value ($\sigma^2 = 1$) throughout the simulations. Then, after introducing the latent variables $\boldsymbol{\tau} = \begin{bmatrix}\tau_1 \cdots \tau_p\end{bmatrix}^{\text{T}}$, we have the following conditional distributions for $\boldsymbol{\beta}$ and $\boldsymbol{\tau}$:

$$\begin{aligned}
\boldsymbol{\beta}|\mathbf{Y}, \sigma^2, \boldsymbol{\tau} &\sim \text{N}(\mathbf{A}^{-1}\mathbf{X}^{\text{T}}\mathbf{Y}, \sigma^2\mathbf{A}^{-1}) \\
(\boldsymbol{\tau} - \mathbf{1})|\mathbf{Y}, \sigma^2, \boldsymbol{\beta} &\sim \prod_{j=1}^{p} \text{GIG}\left(\frac{1}{2}, \psi = \frac{\lambda_1^2}{4\lambda_2\sigma^2}, \chi_j = \frac{\lambda_2\beta_j^2}{\sigma^2}\right),
\end{aligned}$$

where $\mathbf{A} = \mathbf{X}^{\text{T}}\mathbf{X} + \lambda_2\text{diag}[\tau_j/(\tau_j - 1)]$, and GIG denotes the generalized inverse Gaussian distribution.

2.1 Marginal likelihood from Gibbs samples

According to Chib (1995), the log marginal likelihood of a Bayesian model may be calculated from the converged Gibbs samples as:

$$\log m(\mathbf{Y}) = \log\left[\frac{f(\mathbf{Y}|\boldsymbol{\beta}^*)\pi(\boldsymbol{\beta}^*)}{p(\boldsymbol{\beta}^*|\mathbf{Y})}\right] \approx \left[\log\frac{f(\mathbf{Y}|\boldsymbol{\beta}^*)\pi(\boldsymbol{\beta}^*)}{K^{-1}\sum_{k=1}^{K} p(\boldsymbol{\beta}^*|\mathbf{Y}, \boldsymbol{\tau}^{(k)})}\right], \tag{1}$$

where $\boldsymbol{\beta}^*$ is some high posterior density point of $p(\boldsymbol{\beta}|\mathbf{Y})$ and $\boldsymbol{\tau}^{(k)}$ are Gibbs samples indexed by $k = 1, \ldots, K$. In principle, any point $\boldsymbol{\beta}^*$ may be used, but for the sake of efficiency a high-density point of $\boldsymbol{\beta}$ is preferred, such as the posterior mode. Then, for fixed σ^2, the log marginal likelihood is approximated by:

$$\log m(\mathbf{Y}) \approx \left[\log \frac{f(\mathbf{Y}|\boldsymbol{\beta}^*)\pi(\boldsymbol{\beta}^*)}{K^{-1}\sum_{k=1}^K p(\boldsymbol{\beta}^*|\mathbf{Y}, \boldsymbol{\tau}^{(k)})}\right] = \log K - \log\left[\sum_{k=1}^K \frac{p(\boldsymbol{\beta}^*|\mathbf{Y}, \boldsymbol{\tau}^{(k)})}{f(\mathbf{Y}|\boldsymbol{\beta}^*)\pi(\boldsymbol{\beta}^*)}\right]$$

$$= \log K - \log\Bigg\{\sum_{k=1}^K \exp\Bigg[\frac{n-p}{2}\log(2\pi) + \frac{n}{2}\log\sigma^2 + \frac{p}{2}\log 4 - \frac{n}{2}\log\lambda_2$$

$$- p\log\phi\left(\frac{\lambda_1}{\sqrt{4\lambda_2\sigma^2}}\right) + p\log\Phi\left(\frac{-\lambda_1}{\sqrt{4\lambda_2\sigma^2}}\right) + \frac{\lambda_1}{2\sigma^2}\sum_{j=1}^p |\beta_j^*| - \frac{\lambda_2}{2\sigma^2}\sum_{j=1}^p \frac{(\beta_j^*)^2}{\tau_j^{(k)} - 1}$$

$$+ \frac{1}{2}\sum_{j=1}^p \log\left(\frac{\tau_j^{(k)}}{\tau_j^{(k)} - 1}\right) + \frac{1}{2}\log\left|\lambda_2\mathbf{I}_n + \mathbf{X}(\mathbf{I}_p - (\mathbf{T}^{(k)})^{-1})\mathbf{X}^{\mathrm{T}}\right|$$

$$+ \frac{\lambda_2}{2\sigma^2}\mathbf{Y}^{\mathrm{T}}\left(\lambda_2\mathbf{I}_n + \mathbf{X}(\mathbf{I}_p - (\mathbf{T}^{(k)})^{-1})\mathbf{X}^{\mathrm{T}}\right)^{-1}\mathbf{Y}\Bigg]\Bigg\},$$

where $\mathbf{T}^{(k)} = \mathrm{diag}(\tau_j^{(k)})$.

Sampling from the multivariate normal is a costly operation in high dimensions. In Bhattacharya et al. (2015) an efficient sampling scheme for $\boldsymbol{\beta}$ is described:

Set $\mathbf{D} = \lambda_2^{-1}\mathbf{I}_p + \lambda_2^{-1}\mathrm{diag}(\tau_j^{-1})$
Generate $\mathbf{u} \sim \mathrm{N}(\mathbf{0}, \mathbf{D})$
Generate $\mathbf{v} \sim \mathrm{N}(\mathbf{0}, \mathbf{I}_n)$
return $\boldsymbol{\beta} = \mathbf{u} + \mathbf{D}\mathbf{X}^{\mathrm{T}}(\mathbf{X}\mathbf{D}\mathbf{X}^{\mathrm{T}} + \mathbf{I}_n)^{-1}(\mathbf{Y} - \mathbf{X}\mathbf{u} + \mathbf{l})$.

Furthermore, if $(\tau_j - 1)|\mathbf{Y}, \sigma^2, \beta_j \sim \mathrm{GIG}(1/2, \psi, \chi_j)$, then $1/(\tau_j - 1)|\mathbf{Y}, \sigma^2, \beta_j \sim \mathrm{IGauss}(\mu_j = \sqrt{\psi/\chi_j}, \lambda = \psi)$. Sampling from this inverse Gaussian is done by the following scheme:

Generate $U \sim \mathrm{U}(0, 1)$
Generate $Y \sim \mathrm{N}(0, 1)$
Set $z = \sqrt{\frac{\psi}{\chi_j}} + \frac{y^2}{2\chi_j} - \sqrt{\frac{\sqrt{\psi}y^2}{\chi_j^{1\frac{1}{2}}} + \frac{y^4}{4\chi_j^2}}$
if $u \leq (1 + z\sqrt{\chi_j/\psi})^{-1}$ **then**
  return $\tau_j = 1/z + 1$
else
  return $\tau_j = \chi_j z/\psi + 1$.
end if

3 Proof for Theorem 1: EMSE τ^2 for linear regression

First, write

$$\mathrm{EMSE}(\hat{\tau}^2) = E_{\mathbf{X}}\Big[E_{\boldsymbol{\beta}}\big[\mathrm{MSE}(\hat{\tau}^2|\boldsymbol{\beta}, \mathbf{X})\big]\Big] = E_{\mathbf{X}}\Big[E_{\boldsymbol{\beta}}\big[(E_{\mathbf{Y}}(\hat{\tau}^2|\boldsymbol{\beta}, \mathbf{X}) - \tau^2)^2 + V_{\mathbf{Y}}(\hat{\tau}^2|\boldsymbol{\beta}, \mathbf{X})\big]\Big]. \quad (2)$$

Then, let us first compute the expected squared bias w.r.t. $\boldsymbol{\beta}$:

$$
\begin{aligned}
E_{\boldsymbol{\beta}}(\text{bias}^2) &= E_{\boldsymbol{\beta}}[(E_{\mathbf{Y}}(\hat{\tau}^2|\boldsymbol{\beta},\mathbf{X}) - \tau^2)^2] = p^{-2}E_{\boldsymbol{\beta}}[(\sum_{j=1}^{p}(E_{\mathbf{Y}}(\hat{\beta}_j^2) - v_j) - p\tau^2)^2] \\
&= p^{-2}E_{\boldsymbol{\beta}}[(\sum_{j=1}^{p}\beta_j^2 - p\tau^2)^2] = p^{-2}E_{\boldsymbol{\beta}}[(\sum_{j=1}^{p}\beta_j^2)^2 - 2p\tau^2\sum_{j=1}^{p}\beta_j^2 + p^2\tau^4] \\
&= p^{-2}(\sum_{j=1}^{p}3\tau^4 + 2\sum_{j,k\neq j}\tau^4 - p^2\tau^4) = p^{-2}(3p\tau^4 + p(p-1)\tau^4 - p^2\tau^4) \\
&= \frac{2\tau^4}{p},
\end{aligned}
$$

where we used the central moments of Gaussian random variables, available from Isserlis' Theorem (Isserlis, 1918): $E(\beta_j^4) = 3\tau^4$ and $E(\beta_j^2\beta_k^2) = E(\beta_j^2)E(\beta_k^2) = \tau^4$. The result is constant in $\mathbf{X}$, so

$$
E_{\mathbf{X}}[E_{\boldsymbol{\beta}}(\text{bias}^2)] = \frac{2\tau^4}{p}. \tag{3}
$$

Next, we compute $E_{\mathbf{X}}[E_{\boldsymbol{\beta}}[V_{\mathbf{Y}}(\hat{\tau}^2|\boldsymbol{\beta},\mathbf{X})]]$. Denoting $V(\hat{\tau}^2) = V_{\mathbf{Y}}(\hat{\tau}^2|\boldsymbol{\beta},\mathbf{X})$, we have:

$$
V(\hat{\tau}^2) = V((\tau')^2) = \frac{1}{p^2}\Bigg(\sum_{j=1}^{p}V(\hat{\beta}_j^2) + \sum_{j,k\neq j}^{p}\text{Cov}(\hat{\beta}_j^2,\hat{\beta}_k^2)\Bigg). \tag{4}
$$

Hence, we need to compute $V(\hat{\beta}_j^2)$ and $\text{Cov}(\hat{\beta}_j^2,\hat{\beta}_k^2)$. These are again derived from expressions of the central moments of Gaussian random variables. Let us first express the non-central moments in $\text{Cov}_{\mathbf{Y}}(\hat{\beta}_j^2,\hat{\beta}_k^2) = \text{Cov}(\hat{\beta}_j^2,\hat{\beta}_k^2) = E(\hat{\beta}_j^2\hat{\beta}_k^2) - E(\hat{\beta}_j^2)E(\hat{\beta}_k^2)$ in terms of the central ones. Denote the centralized value of $\hat{\beta}_j$ by $\tilde{\beta}_j = \hat{\beta}_j - \beta_j$. Then,

$$
\begin{aligned}
E[\hat{\beta}_j^2\hat{\beta}_k^2] &= E[((\hat{\beta}_j - \beta_j) + \beta_j)^2((\hat{\beta}_k - \beta_k) + \beta_k)^2] = E[(\tilde{\beta}_j + \beta_j)^2(\tilde{\beta}_k + \beta_k)^2] \\
&= T_1 + T_2 := E[\tilde{\beta}_j^2\tilde{\beta}_k^2 + 4\beta_j\beta_k\tilde{\beta}_j\tilde{\beta}_k + \beta_j^2\beta_k^2 + \tilde{\beta}_j^2\beta_k^2 + \beta_j^2\tilde{\beta}_k^2] \\
&+ E[2\beta_j\tilde{\beta}_j\tilde{\beta}_k^2 + 2\beta_k\tilde{\beta}_k\tilde{\beta}_j^2 + 2\beta_j\tilde{\beta}_j\beta_k^2 + 2\beta_k\tilde{\beta}_k\beta_j^2] \\
&= T_1,
\end{aligned}
$$

because $T_2 = 0$ due to the symmetry of the central Gaussian distribution. Likewise, the second term of the covariance equals:

$$
E(\hat{\beta}_j^2)E(\hat{\beta}_k^2) = E(\tilde{\beta}_j^2)E(\tilde{\beta}_k^2) + \beta_j^2E(\tilde{\beta}_k^2) + \beta_k^2E(\tilde{\beta}_j^2) + \beta_j^2\beta_k^2.
$$

Subtracting the latter from T_1 cancels the latter 3 terms in both expressions, rendering

$$
\begin{aligned}
\text{Cov}(\hat{\beta}_j^2,\hat{\beta}_k^2) &= E(\tilde{\beta}_j^2\tilde{\beta}_k^2) + 4\beta_j\beta_kE(\tilde{\beta}_j\tilde{\beta}_k) - E(\tilde{\beta}_j^2)E(\tilde{\beta}_k^2) \\
&= (v_jv_k + 2v_{jk}^2) + 4\beta_j\beta_kv_{jk} - v_jv_k = 2v_{jk}^2 + 4\beta_j\beta_kv_{jk},
\end{aligned} \tag{5}
$$

where we used the equations for the central moments of Gaussian random variables (Isserlis, 1918). Noting that $V(\hat{\beta}_j^2) = \text{Cov}(\hat{\beta}_j^2,\hat{\beta}_j^2)$ we directly obtain

$$
\begin{aligned}
V(\hat{\beta}_j^2) &= E(\tilde{\beta}_j^4) + 4\beta_j\beta_jE(\tilde{\beta}_j^2) - (E(\tilde{\beta}_j^2))^2 \\
&= 3v_j^2 + 4\beta_j^2v_j - v_j^2 = 2v_j^2 + 4\beta_j^2v_j.
\end{aligned} \tag{6}
$$

Note that the latter can also be obtained by writing $\hat{\beta}_j^2 = v_j(\hat{\beta}_j/\sqrt(v_j))^2 = v_j(\beta'_j)^2$. Then $\beta'_j \sim N(\beta_j/\sqrt(v_j), 1)$, so $(\beta'_j)^2 \sim \chi^2(\nu = \beta_j^2/v_j, k = 1)$ with $V((\beta'_j)^2) = 2(k + 2\nu) = 2(1 + 2\beta_j^2/v_j)$. Hence, indeed $V(\hat{\beta}_j^2) = v_j^2 V((\beta'_j)^2) = 2v_j^2 + 4\beta_j^2 v_j$.
Substituting (5) and (6) into (4) renders:

$$V(\hat{\tau}^2) = \frac{2}{p^2}[\sum_{j=1}^{p}(v_j^2 + 2\beta_j^2 v_j) + \sum_{j,k\neq j}(v_{jk}^2 + 2\beta_j\beta_k v_{jk})].$$

Taking expectation w.r.t. $\boldsymbol{\beta}$ gives:

$$E_{\boldsymbol{\beta}}[V(\hat{\tau}^2)] = \frac{2}{p^2}[\sum_{j=1}^{p}(v_j^2 + 2\tau^2 v_j) + \sum_{j,k\neq j}^{p} v_{jk}^2],$$

because we assume i.i.d. central priors for β_j. Now to compute

$$E_{\mathbf{X}}[E_{\boldsymbol{\beta}}[V(\hat{\tau}^2)]] = \frac{2}{p^2}[\sum_{j=1}^{p}(E_{\mathbf{X}}(v_j^2) + 2\tau^2 E_{\mathbf{X}}(v_j)) + \sum_{j,k\neq j}^{p} E_{\mathbf{X}}(v_{jk}^2)] \tag{7}$$

we need to know $E_{\mathbf{X}}(v_j^2), E_{\mathbf{X}}(v_j)$ and $E_{\mathbf{X}}(v_{jk}^2)$, where $v_j = v_{jj}$ and $v_{jk} = V_{jk}, V = (\mathbf{X}^T\mathbf{X})^{-1}$, with $X_i \sim N(0, \Sigma = \Sigma_{p\times p})$ and $\Sigma_{jj} = 1$. By definition, V follows an inverse-Wishart distribution: $V \sim \mathcal{W}^{-1}(\Psi = \Sigma^{-1}, n)$. Hence, the requested moments are known (Press, 1982):

$$\begin{aligned}
&E_{\mathbf{X}}(v_j) = \psi_{jj}/(n-p-1)\\
&E_{\mathbf{X}}(v_j^2) = V_{\mathbf{X}}(v_j) + (E_{\mathbf{X}}(v_j))^2 = \frac{2\psi_{jj}^2}{(n-p-1)^2(n-p-3)} + \frac{\psi_{jj}^2}{(n-p-1)^2}\\
&E_{\mathbf{X}}(v_{jk}^2) = V_{\mathbf{X}}(v_{jk}) + (E_{\mathbf{X}}(v_{jk}))^2 = \frac{(n-p+1)\psi_{jk}^2 + (n-p-1)\psi_{jj}\psi_{kk}}{(n-p)(n-p-1)^2(n-p-3)} + \frac{\psi_{jk}^2}{(n-p-1)^2},
\end{aligned} \tag{8}$$

where we assume $p < n-3$. Substituting (8) into (7) and aggregating with the expected squared bias (3) finalizes the result:

$$\begin{aligned}
\text{EMSE}(\hat{\tau}^2) = \frac{2}{(n-p-1)p^2}\Bigg[&\sum_{j=1}^{p}\Big(\frac{2\psi_{jj}^2}{(n-p-1)(n-p-3)} + \frac{\psi_{jj}^2}{(n-p-1)}\Big) + 2\tau^2\sum_{j=1}^{p}\psi_{jj}\\
&+ \sum_{j,k\neq j}^{p}\Big(\frac{(n-p+1)\psi_{jk}^2 + (n-p-1)\psi_{jj}\psi_{kk}}{(n-p)(n-p-1)(n-p-3)} + \frac{\psi_{jk}^2}{(n-p-1)}\Big)\Bigg] + \frac{2\tau^4}{p}.
\end{aligned} \tag{9}$$

This simplifies for independent X_i, because then $\psi_{jj} = 1$ and $\psi_{jk} = 0$:

$$\begin{aligned}
\text{EMSE}_{\perp}(\hat{\tau}^2) = \frac{2}{(n-p-1)p}\Bigg[&\frac{2}{(n-p-1)(n-p-3)} + \frac{1}{n-p-1} + 2\tau^2\\
&+ \frac{p-1}{(n-p)(n-p-3)}\Bigg] + \frac{2\tau^4}{p}.
\end{aligned} \tag{10}$$

4 Supporting Figures

4.1 Baseball Example

4.2 Simulation Example

Here, we show the results for all simulation settings presented in the Simulation example.

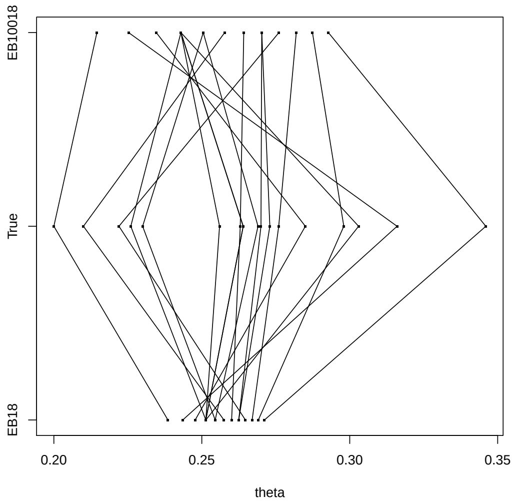

(a) 18 vs 10018

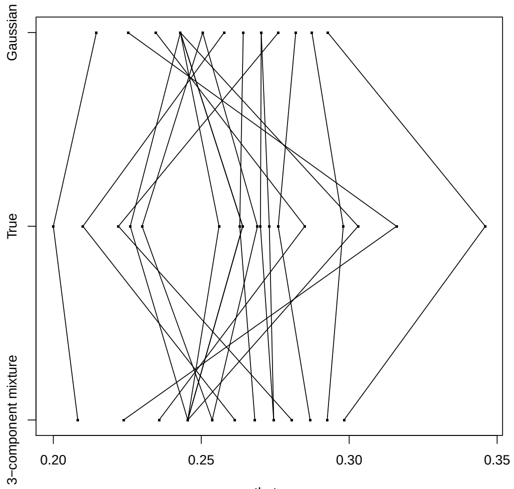

(b) Gaussian vs Mixture

Figure 1: Shrinkage estimators vs true value. Left: Parameter estimates for 18 real players when EB estimation of hyper-parameters is based on either 18 (bottom) or 10018 (top) players. Right: Parameter estimates for 18 real players when EB estimation of hyper-parameters is based on 10018 players, using as prior either a 3-component mixture of Gaussians (bottom) or a Gaussian (top).

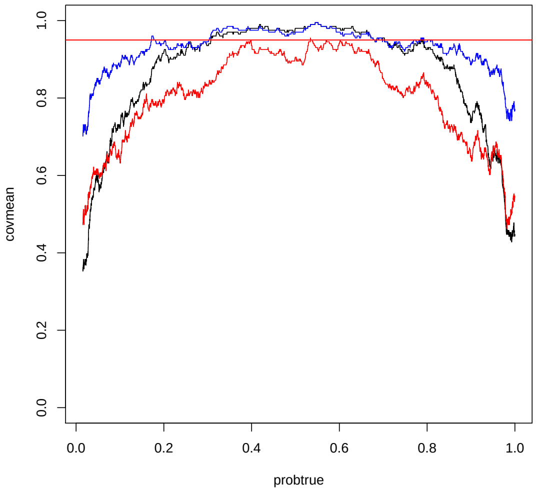

(a) Equal-tailed interval

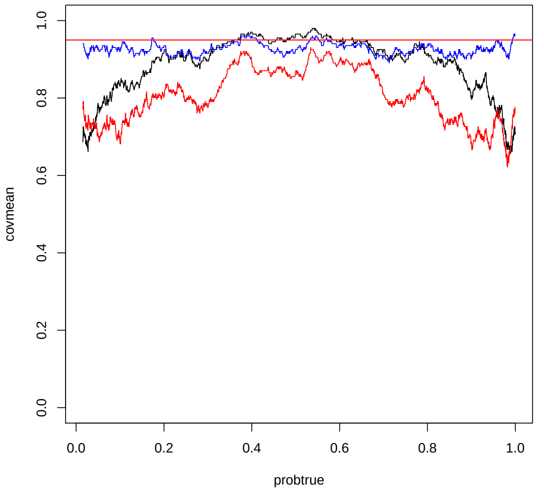

(b) HPD interval

Figure 2: X-axis: True event probability q_j; Y-axis: mean coverage of 95% posterior intervals for event probability. Mean is estimated by moving average. Case: $G = 2, p_g = 10, p = G * p_G = 20, n_{\text{train}} = 100$. Methods: Hyb, EB, FB

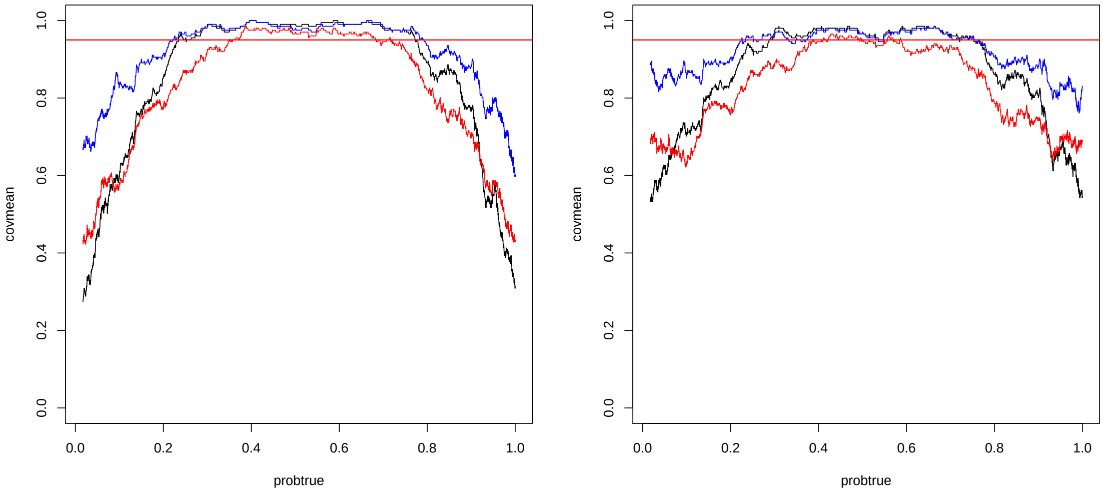

(a) Equal-tailed interval

(b) HPD interval

Figure 3: X-axis: True event probability q_j; Y-axis: mean coverage of 95% posterior intervals for event probability. Mean is estimated by moving average. Case: $G = 2, p_g = 20, p = G * p_G = 40, n_{\text{train}} = 100$. Methods: Hyb, EB, FB

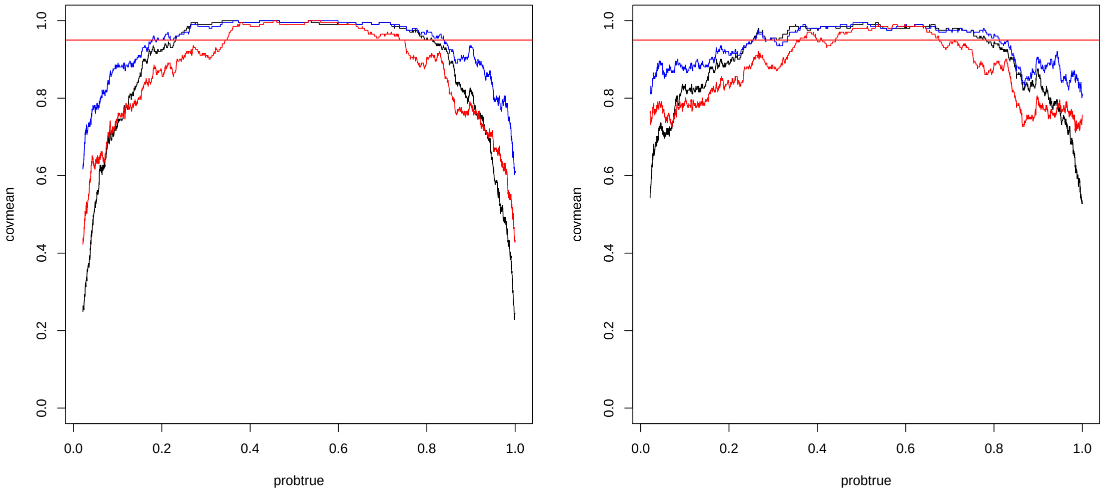

(a) Equal-tailed interval

(b) HPD interval

Figure 4: X-axis: True event probability q_j; Y-axis: mean coverage of 95% posterior intervals for event probability. Mean is estimated by moving average. Case: $G = 2, p_g = 30, p = G * p_G = 60, n_{\text{train}} = 100$. Methods: Hyb, EB, FB

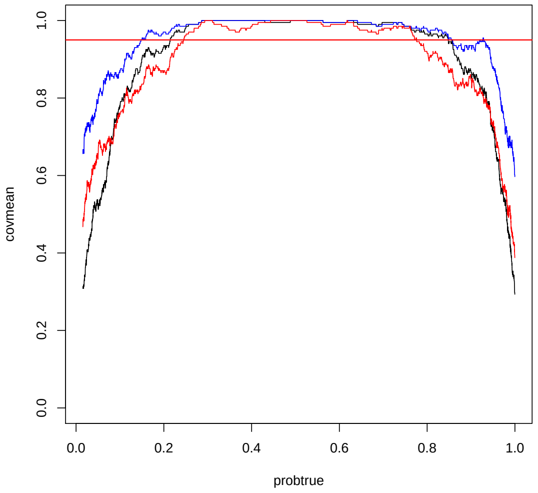

(a) Equal-tailed interval

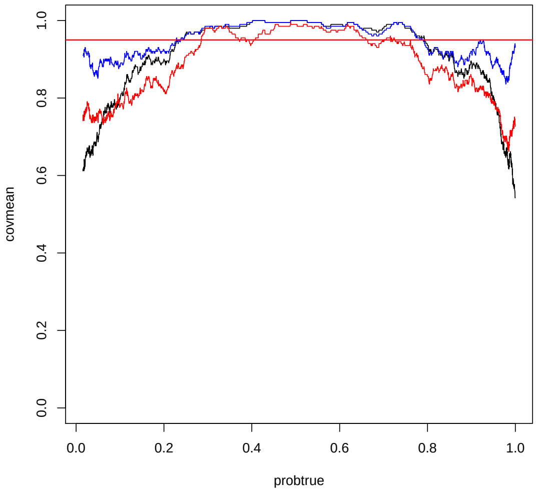

(b) HPD interval

Figure 5: X-axis: True event probability q_j; Y-axis: mean coverage of 95% posterior intervals for event probability. Mean is estimated by moving average. Case: $G = 2, p_g = 40, p = G * p_G = 80, n_{\text{train}} = 100$. Methods: Hyb, EB, FB

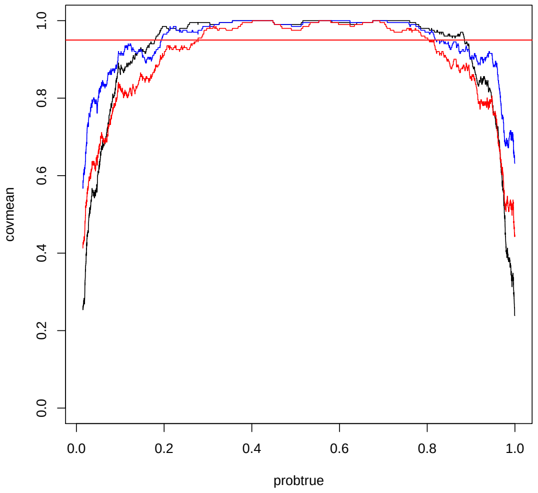

(a) Equal-tailed interval

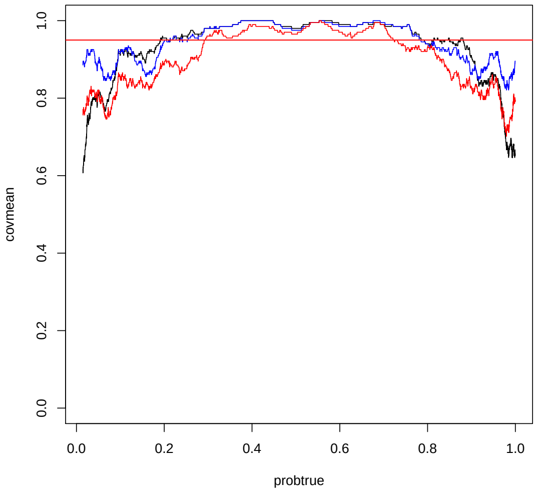

(b) HPD interval

Figure 6: X-axis: True event probability q_j; Y-axis: mean coverage of 95% posterior intervals for event probability. Mean is estimated by moving average. Case: $G = 2, p_g = 50, p = G * p_G = 50, n_{\text{train}} = 100$. Methods: Hyb, EB, FB

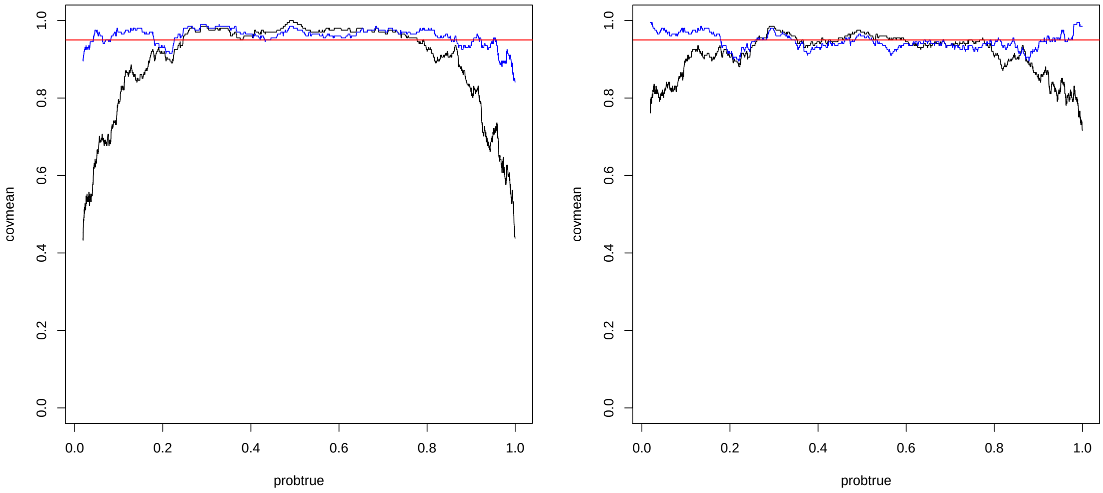

(a) Equal-tailed interval

(b) HPD interval

Figure 7: X-axis: True event probability q_j; Y-axis: mean coverage of 95% posterior intervals for event probability. Mean is estimated by moving average. Case: $G = 5, p_g = 10, p = G * p_G = 50, n_{\text{train}} = 200$. Methods: Hyb, EB

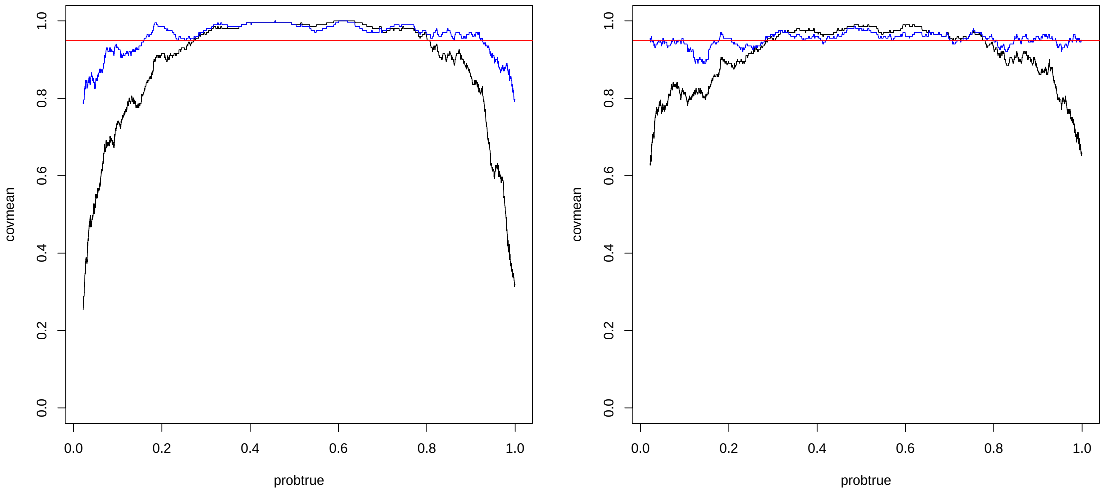

(a) Equal-tailed interval

(b) HPD interval

Figure 8: X-axis: True event probability q_j; Y-axis: mean coverage of 95% posterior intervals for event probability. Mean is estimated by moving average. Case: $G = 5, p_g = 20, p = G * p_G = 100, n_{\text{train}} = 200$. Methods: Hyb, EB

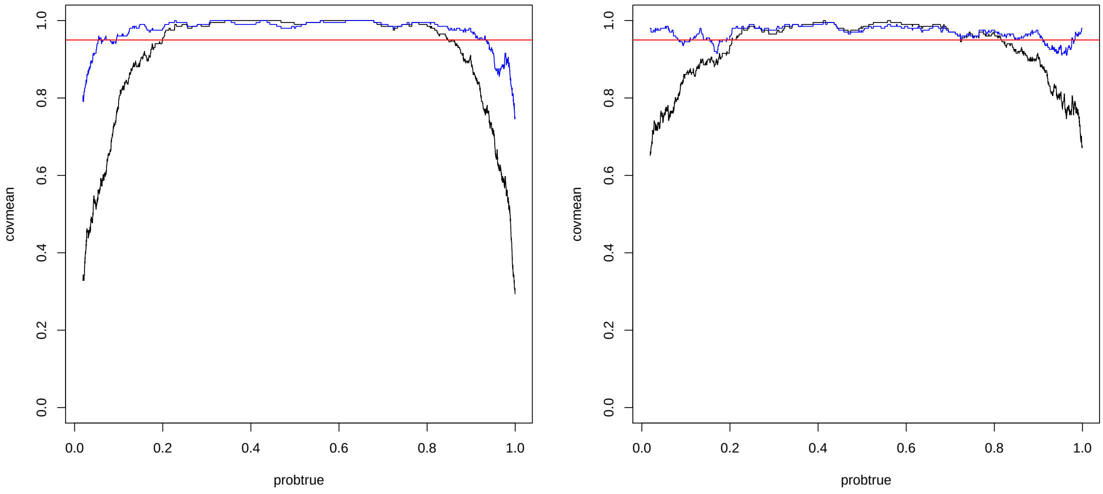

(a) Equal-tailed interval

(b) HPD interval

Figure 9: X-axis: True event probability q_j; Y-axis: mean coverage of 95% posterior intervals for event probability. Mean is estimated by moving average. Case: $G = 5, p_g = 40, p = G * p_G = 200, n_{\text{train}} = 200$. Methods: Hyb, EB

References

Bhattacharya, A. et al. (2015). Fast sampling with Gaussian scale-mixture priors in high-dimensional regression. *arXiv:1506.04778 [stat]*. ArXiv: 1506.04778.
URL `http://arxiv.org/abs/1506.04778`

Chib, S. (1995). Marginal likelihood from the Gibbs output. *J. Amer. Statist. Assoc.*, **90**, 1313–1321.

Efron, B. and Morris, C. (1975). Data analysis using Stein's estimator and its generalizations. *J. Amer. Statist. Assoc.*, **70**, 311–319.

Isserlis, L. (1918). On a formula for the product-moment coefficient of any order of a normal frequency distribution in any number of variables. *Biometrika*, **12**, 134–139.

Li, Q. and Lin, N. (2010). The Bayesian elastic net. *Bayesian Analysis*, **5**, 151–170.

Press, S.J. (1982). *Applied Multivariate Analysis, 2nd ed.* Dover Publications, New York.

Van de Wiel, M.A. et al. (2012). Bayesian analysis of RNA sequencing data by estimating multiple shrinkage priors. *Biostatistics*, **14**, 113–128.

Van Houwelingen, H.C. (2014). The role of empirical Bayes methodology as a leading principle in modern medical statistics. *Biom. J.*, **56**, 919–932.
